# Single-cell RNA-Seq reveals the transcriptional landscape and heterogeneity of skin macrophages in Vsir^-/-^ murine psoriasis

**DOI:** 10.7150/thno.45614

**Published:** 2020-08-21

**Authors:** Chenxin Qie, Jingwei Jiang, Wanmei Liu, Xinlei Hu, Wenting Chen, Xiaoxue Xie, Jun Liu

**Affiliations:** 1 Jiangsu key lab of Drug Screening, China Pharmaceutical University, Nanjing, 210009, China.; 2 Nanjing Gemini Biotechnology Co. Ltd, Nanjing, 210009, China.

**Keywords:** Single-cell RNA-sequencing, psoriasis, imiquimod, VISTA, macrophages

## Abstract

**Rationale:** V-domain immunoglobulin suppressor of T cell activation (VISTA) is a novel inhibitory immune checkpoint molecule. Vsir^-/-^ mice have exacerbated psoriasis-like skin inflammation. The immune cell subsets involved in inflammation in Vsir^-/-^ psoriatic mice are largely unknown. We have used scRNA-seq as an unbiased profiling strategy to study the heterogeneity of immune cells at a single cell level in the skin of Vsir^-/-^ psoriatic mice.

**Methods:** In the present study, the right ear and shaved back skin of wild type and Vsir^-/-^ mice were treated with IMQ for 5 consecutive days to induce psoriasis-like dermatitis. Then, the single-cell RNA sequencing analysis of mouse back skin lesions was performed using 10 × Genomics technique.

**Results:** We identified 12 major cell subtypes among 23,258 cells. The major populations of the skin cells included macrophages, dendritic cells and fibroblasts. Macrophages constituted the main immune cell population in the WT (61.29%) and Vsir^-/-^ groups (77.7%). It should be noted that DCs and fibroblasts were expanded in the Vsir^-/-^ psoriatic mice. Furthermore, the gene expression signatures were assessed. We observed that Hspb1 and Cebpb were significantly upregulated in the Vsir^-/-^ psoriatic mice. Differential gene expression and gene ontology enrichment analyses revealed specific gene expression patterns distinguishing these subsets and uncovered putative functions of each cell type. Date analysis resulted in the discovery of a number of novel psoriasis-associated genes in Vsir^-/-^ mice.

**Conclusion:** We present a comprehensive single-cell landscape of the skin immune cells in Vsir^-/-^ psoriatic mice. These unprecedented data uncovered the transcriptional landscape and phenotypic heterogeneity of skin macrophages in psoriasis and identified their gene expression signature suggesting specialized functions in Vsir^-/-^ mice. Our findings will open novel opportunities to investigate the role of VISTA in driving psoriasis.

## Introduction

Psoriasis is one of the most common autoimmune skin diseases 1 affecting approximately 2% to 3% of the population worldwide. The multifactorial pathogenesis contributing to the psoriasis phenotype involves genetic, environmental and immunological factors 2. Psoriasis is generally manifested as chronic inflammation of the skin and is characterized by circumscribed, scaling, and erythaematous plaques 3, 4.

V-domain immunoglobulin suppressor of T cell activation (VISTA) [also called programmed death-1 homologue (PD-1H), VSIR (V-set immunoregulatory receptor), Dies1, DD1α, and Gi24] is an inhibitory B7 family immune-checkpoint molecule expressed on T cells and myeloid cells 5. Previous studies have reported that VISTA functions as an inhibitory ligand on myeloid cells and also as an inhibitory receptor on T cells 6-8. It should be noted that VISTA has been shown to negatively regulate immune function in a variety of autoimmune diseases. Vsir knockout mice (Vsir^-/-^) develop loss of peripheral tolerance, manifested as spontaneous T cell activation, production of inflammatory cytokines and chemokines, and chronic multi-organ inflammation 9. VISTA-deficient mice are more sensitive to experimental autoimmune encephalomyelitis (EAE) characterized by an increase in IFN-γ^+^ and IL-17A^+^ producing CD4^+^ T cells detected in the central nervous system (CNS) 10. VISTA-deficient mice on a BALB/c background develop more severe arthritis and lupus 11. Application of a VISTA-blocking antibody (13F3) significantly accelerated disease progression and exacerbated disease severity in a mouse model of multiple sclerosis 12. Imiquimod (IMQ) is a known ligand of toll-like receptors 7 (TLR7) and 8 (TLR8). A sole application of IMQ rapidly induces dermatitis closely resembling human psoriasis 13-15. In IMQ-induced model of psoriasis, VISTA deficiency augmented the inflammatory responses, resulting in exacerbated psoriasiform dermatitis 16. Irrespective of these findings, the role of VISTA in psoriasis is largely unknown.

Skin from psoriatic patients is characterized by a dense dermal infiltrate that predominantly consists of T-cells, dendritic cells (DCs), natural killer T-cells, and macrophages. Subsets of human and mouse monocytes can develop into macrophages in various disease states 17, 18. Psoriasis has been regarded as a T-cell-mediated disease 19-22. However, an increasing body of correlative evidence has shown that macrophages may also be relevant for pathophysiology of psoriasis 23-25. The macrophages are important for ongoing inflammation within the psoriasis and maybe an important target for novel therapeutic strategies in patients with psoriasis. Similar to psoriasis, activated macrophages were found to play the major roles in other T-cell-mediated autoimmune diseases such as rheumatoid arthritis 26.

Over the past ten years, the rapid development of new technologies has enabled us to quickly obtain a large number of physiological and pathological information of psoriasis 27. The advent of single-cell RNA sequencing (scRNA-seq) has enabled the profiling of specific cell populations at a single-cell level. Notably, scRNA-seq has been widely used to reveal the characteristics of immunity in various fields because it can detect changes in individual cell types 28, 29. Here, we performed scRNA-seq of total skin cells extracted from C57BL/6J wild type (WT) and Vsir^-/-^ psoriatic mice induced by IMQ. These unprecedented data uncovered the transcriptional landscape and phenotypic heterogeneity of skin macrophages in psoriasis and identified their gene expression signature, suggesting specialized functions.

## Materials and Methods

### Mice and treatments

Six- to seven-week-old female wild type C57BL/6J mice (Beijing Vital River Laboratory Animal Technologies Co. Ltd) and Vsir^-/-^ mice (C57BL/6J background, obtained from the Shanghai Model Organisms Center, Inc.) were bred and housed under specific pathogen-free conditions. The Vsir^-/-^ allele has been described previously 7. The mice were allowed to acclimate in our facility for one week before the experiments. All animal experiments were performed in accordance with the Laboratory Animal Management Committee of Jiangsu Province and approved by the ethics committee of China Pharmaceutical University.

### IMQ-induced psoriasis-like skin inflammation

Wild type and Vsir^-/-^ mice received a daily topical dose of 62.5 mg of commercially available IMQ cream (5%) (Imiquimod cream; Sichuan Med-Shine Pharmacetical Co. Ltd) on the shaved back skin and right ear for five consecutive days. At the indicated days, the thickness of the right ear was measured in duplicate using a micrometer. The data were used to evaluate epidermal inflammation and proliferation.

### Skin cells isolation

Back lesion skin samples from WT or Vsir^-/-^ psoriatic mice were pooled to generate samples for isolation of viable cells. The back skins were carefully separated from muscle tissues, minced and digested for 90 min at 37 °C in RPMI containing 0.28 mg/mL Liberase TM (Roche) and 0.2 mg/mL DNase (Sigma-Aldrich). The digested skins were passed through a 40 μm cell strainer to obtain single cell suspensions.

### Histological analysis

For histopathological analysis, hematoxylin-eosin (H&E) staining was performed on formaldehyde fixed, paraffin-embedded skin samples. Microscopic analysis of the staining was evaluated by examining five sections from each mouse. The sections were observed using a laser scanning confocal microscope. The thickness of the epidermis was quantified using the ImageJ software.

### Immunohistochemistry (IHC)

Tissues were fixed in 10% neutral buffered, formalin and paraffin embedded and standard IHC was performed using rabbit anti-CD3 pAb (1:150 dilution; GB13014; Servicebio). Serial sections were stained with rabbit anti-CD68 pAb (1:500 dilution; GB11067, Servicebio) and rabbit anti-LY6G pAb (1:500 dilution; GB11229, Servicebio) and visualized using peroxidase/DAB. Negative controls were included by incubation of the sections only with a secondary antibody. Quantification was performed by counting positive cells in 6 to 10 high-powered fields (magnification, ×200) in a blinded fashion.

### Single cell RNA-seq protocol and data analysis

Single cells were encapsulated in droplets using 10 × Genomics GemCode Technology and processed according to the manufacturer's instruction. Briefly, every cell and every transcript were uniquely barcoded using a unique molecular identifier (UMI). Libraries were generated and sequenced from the cDNA and the 10 × barcodes were used to associate individual reads back to the individual partitions. The single cell 3' protocol produces Illumina-ready sequencing libraries. Transcripts were mapped to the mm10-3.0.0 reference genome. Cell Ranger uses an aligner called STAR, which peforms splicing-aware alignment of reads to the genome. When combining data from multiple libraries, we recommend equalizing the read depth between libraries before merging to reduce the batch effect introduced by sequencing. Reads from higher-depth libraries were subsampled until they all have an equal number of total reads per cell.

### Quality control metrics and data processing

For downstream analyses after initial Cell Ranger metric assessment, low-quality cells were removed to eliminate cell-specific biases. We utilized the following procedure to control data quality: cells with fewer than 500 genes or more than 4,000 genes detected, cells with more than 80,000 UMIs detected, and cells for which more than 10% of UMIs were derived from mitochondrial genes were excluded. Post-quality control, 23,258 cells remained for downstream bioinformatics analyses. To avoid overclustering, we merged the clusters that were not transcriptionally distinct into a single cluster. Clusters composed of doublets (two different cell types within a single droplet) were removed from the dataset. We used a global-scaling normalization method “LogNormalize” that normalizes the gene expression measurements for each cell by the total expression, multiplies this by a scale factor (10,000 by default), and log-transforms the result. The formula was as follows: A gene expression level=log(1 + (UMIA/UMI Total) × 10000). We implemented a resampling test inspired by the jackStraw procedure. We randomly permute a subset of the data (1% by default) and rerun principal component analysis (PCA) constructing a 'null distribution' of the gene scores and repeat this procedure. Marker genes were determined with *p*-value ≤ 0.01 and log(fold-change) ≥ 0.360674 by performing differential gene expression analysis using the likelihood-ratio test. The percentage of the cells where the gene is detected in a specific cluster is over 25%. Data clustering was performed using the Seurat R package. Cell types for the analysis were derived from the PanglaoDB. The t-distributed stochastic neighbour embedding (tSNE) was used for data visualization in two dimensions. The clusters were obtained with resolution = 0.5.

### Analysis of differentially expressed genes in WT and Vsir^-/-^ mice groups

We used a hurdle model in MAST (Model-based Analysis of Single-cell Transcriptomics) 30 to find differentially expressed genes for a group in one cluster. We identified differentially expressed genes based on the following criteria: |logFC| ≥ 0.25, *p*_value_adj ≤0.05 and the percentage of cells where the gene is detected in specific cluster is more than 25%. We used the gene ontology (GO) enrichment analysis to discover certain biological functions in each cell type. Initially, all peak related genes were mapped to GO terms in the Gene Ontology database; gene numbers were calculated for every term and significantly enriched GO terms in the peak related genes versus to the genome background were defined by hypergeometric test.

### Single cell TCR analysis

GEMs were generated by combining barcoded Single Cell 5'Gel beads, a master mix with cells, and partitioning oil on a microfluidic chip. Barcoded, full-length cDNA was amplified via PCR with primers against common 5' and 3' ends added during GEM-RT. Barcoded, full-length V(D)J segments were enriched from the amplified cDNA via PCR amplification with primers specific to either the TCR or Ig constant regions prior to library construction. Enzymatic fragmentation and size selection were used to generate variable length fragments that collectively span the V(D)J segments of the enriched TCR or Ig transcripts prior to library construction. The single cell V(D)J reagent kit protocol produced V(D)J-enriched and 5' gene expression Illumina-ready sequencing libraries. Analysis pipelines in Cell Ranger were used for sequencing data processing. The raw sequencing data generated from this study have been deposited in NCBI SRA under the accession number SRP 268188 (WT+IMQ) and SRP 250740 (sample KI in this accession number is Vsir^-/-^+IMQ in this study). Other data and analytical methods are available from the corresponding authors upon reasonable request. The cDNA/DNA/small RNA libraries were sequenced on the Illumina sequencing platform by Genedenovo Biotechnology Co., Ltd. (Guangzhou, China).

### Pseudotime Analysis

Single cell trajectory was analysed using matrix of cells and gene expressions by Monocle (Version2.6.4). Monocle reduced the space down to one with two dimensions and ordered the cells (sigma = 0.001, lambda = NULL, param.gamma = 10, tol = 0.001). We identified the key genes related to the development and differentiation process with FDR <1e-5 and grouped the genes with similar trends in expression reasoning that such groups may share common biological functions and regulators. Monocle developed BEAM to test for branch-dependent gene expression by formulating the problem as a contrast between two negative binomial GLMs. Differential gene testing for the pseudo-time analysis was based on the previously identified cell clusters.

### Statistical analysis

Statistical analyses were performed using GraphPad Prism. The data are expressed as the mean ± SEM unless indicated otherwise. Unpaired Student's *t*-test was used to determine statistically significant differences. A value of *P* < 0.05 was considered significant at the 95% confidence level. Date analysis was performed using the OmicShare tools, a free online platform for data analysis.

## Results

### Single-cell RNA-seq identified psoriasis-associated immune cell populations in wild type and Vsir^-/-^ mice

**Figure 1:**
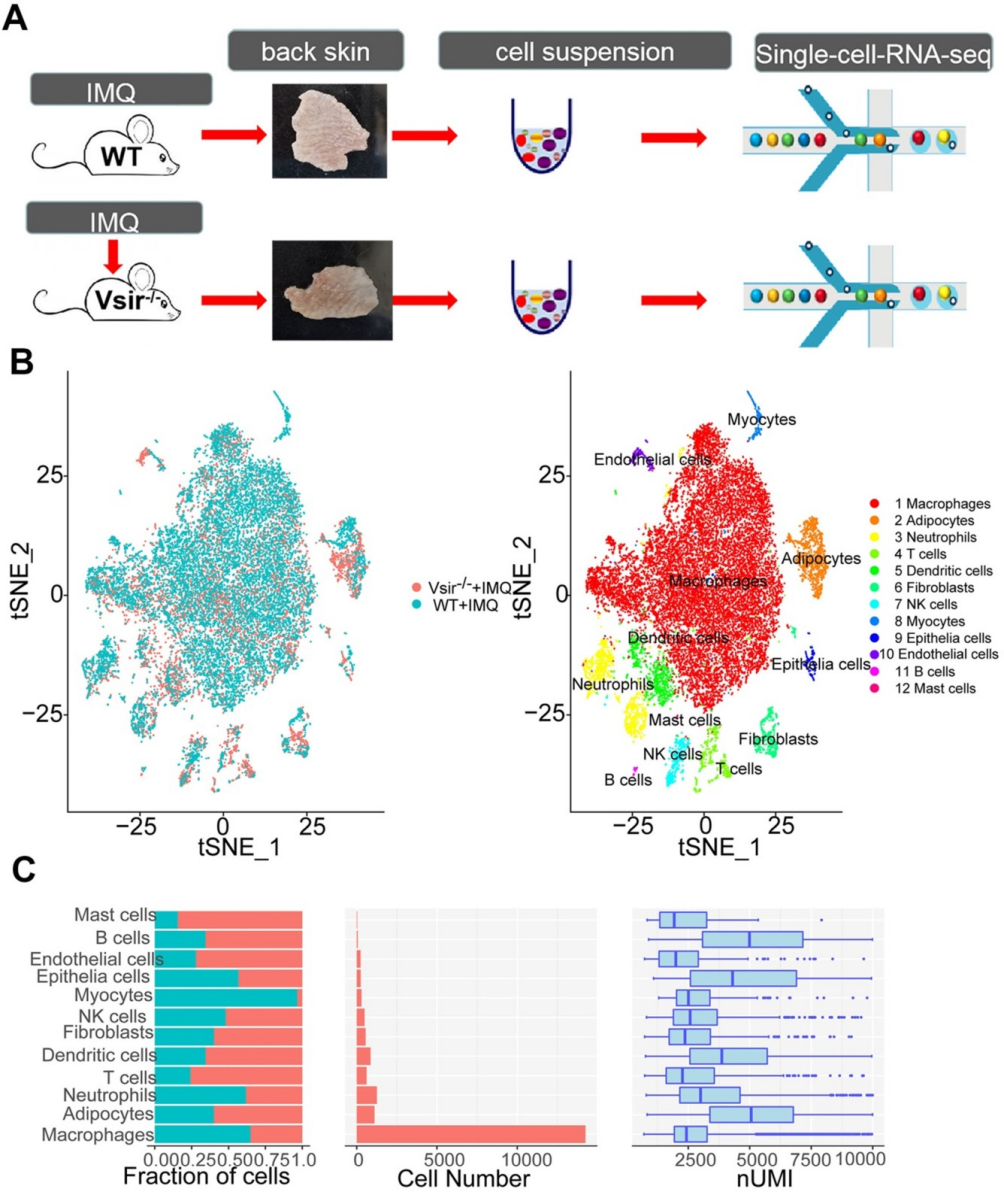
** IMQ-induced psoriasis-associated immune cell populations in WT and Vsir^-/-^ psoriatic mice were identified.** (**A**)Schematic diagram of the experimental design. (**B**) Profiles of the tSNE plots of 23,258 cells extracted from back skin of WT (12,040 cells) and Vsir^-/-^ (11,218 cells) psoriatic mice with each cell colour-coded according to sample origin (left panel) and associated cell type (right panel). (**C**) For each of 12 cell clusters (from left to right), the fraction of cells originating from WT and Vsir^-/-^ psoriatic mice; the number of cells and box plots of the number of transcripts are shown to provide an overview of all immune cells. IMQ, imiquimod. NK cells, natural killer cells. tSNE, t-distributed stochastic neighbour embedding. UMI, unique molecular identifier. Vsir^-/-^, Vsir knockout mice. WT, wild type.

To discover the altered regulation of gene expression in IMQ-induced WT and Vsir^-/-^ psoriatic mice, we performed scRNA-seq of back skin cells from the WT and Vsir^-/-^ mice. We examined IMQ-induced psoriasis in WT and Vsir^-/-^ mice that were topically treated with 5% IMQ on the right ear and back skin. The skin inflammatory response was quantified by measuring the right ear thickness. IMQ treatment in the Vsir^-/-^ mice resulted in more severe ear swelling than that in WT mice (Figure S1A). H&E staining of the right ear skin of the WT and Vsir^-/-^ psoriatic mice validated this conclusion (Figure S1B-C). The back skins in each group were pooled to obtain single cell suspensions. The harvested skin cells from different groups were sequenced on a 10 × Genomics platform (Figure 1A). After application of quality control filters (Figure S2; Table S1), we obtained a total of 23,258 single cell transcriptomes (12,040 WT+IMQ; 11,218 Vsir^-/-^+IMQ) from two pairs of mice.

Our initial goal was to visualize and ultimately define the various cell subsets in the dataset; hence, we analysed the gene expression differences between each single cluster and all other cells to identify the cluster marker genes. Subsequently, we used *t*-distributed stochastic neighbour embedding (tSNE) visualization of the cells to reveal 12 major clusters, including macrophages (7 cell clusters), dendritic cells (DCs; 4 cell clusters), neutrophils (5 cell clusters), T cells (1 cell cluster), fibroblasts (3 cell clusters), epithelial cells (1 cell clusters), natural killer (NK) cells (1 cell cluster), endothelial cells (ECs, 1 cell cluster), mast cells (1 cell cluster), B cells (1 cell cluster), myocytes (1 cell cluster) and adipocytes (1 cell cluster) (Figure 1B). Each cell cluster was derived from different groups and contained a variable number of cells and variable transcriptional activity determined by unique molecular identifiers (UMIs) (Figure 1C). Using the differentially expressed gene signatures, we attributed clusters to their putative identities. We identified the marker genes for each cluster, such as Adgre1 for macrophages, Cd86 for dendritic cells, Mmp8 for neutrophils, Cd3e for T cells and Apod for fibroblasts (Figure S3). Next, we attempted to discern the cellular differences between WT and Vsir^-/-^ psoriatic mouse skin tissue. We found that several clusters of the cells displayed differential composition between the two genotypes. The top 5 immune cell clusters derived from Vsir^-/-^ psoriatic mice were macrophages (61.29%), adipocytes (8.19%), DCs (6.98%), neutrophils (5.91%) and T cells (5.82%; Figure S4B). Macrophages (77.7%) constituted the major populations of immune cells in WT psoriatic mice followed by neutrophils (6.56%), adipocytes (3.73%), DCs (2.52%) and myocytes (2.4%) (Figure S4B). Macrophages are the largest cell population in the WT and Vsir^-/-^ groups. Thus, we decided to focus on the gene expression heterogeneity of the macrophages.

### Gene expression heterogeneity in macrophage subsets was identified in the murine psoriasis

**Figure 2:**
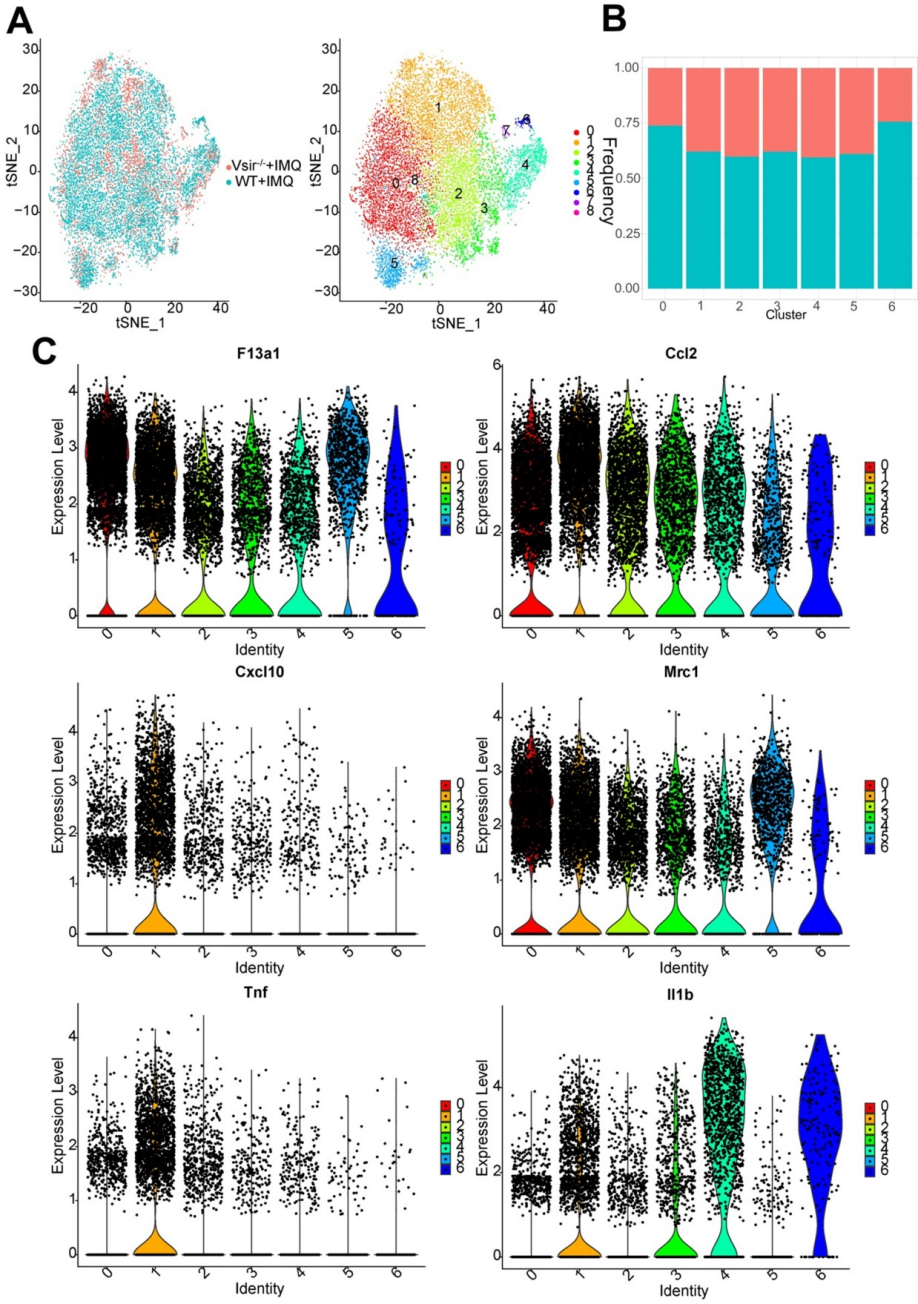
** Signature genes for macrophages in Vsir^-/-^ psoriatic mice were described.** (**A**) The sample origin (left panel) and the tSNE plots of 14,243 macrophages (right panel). Macrophage clusters 7 and 8 come from contaminants. (**B**) Pecentage of macrophage subtypes. Blocks represent different subjects and are colour-coded according to derived groups defined in figure A. (**C**) Violin plots of M1/2 marker genes and specific marker genes in each macrophage cluster. Vsir^-/-^: Vsir knockout mice; WT: wild type.

We detected 14,243 macrophages as the largest cell population (Figure 2A). Macrophages were the major cell population (61.29%) in Vsir^-/-^ psoriatic mice, and approximately 77.7% of skin cells were macrophages in WT psoriatic mice (Figure S4B). Macrophages highly expressed Adgre1 and F13a1 (Figure S3 and Figure 2C). A total pool of macrophages was separated into 7 clusters, namely, macrophage cluster 0 (Ltc4s^+^ Fcna^+^ macrophages), macrophage cluster 1 (Ifit1^+^ Rsad2^+^ macrophages), macrophage cluster 2 (Slamf9^+^ Acp5^+^ macrophages), macrophage cluster 3 (H2-Eb1^+^ H2-Aa^+^ macrophages), macrophage cluster 4 (Ly6c2^+^ Vcan^+^ macrophages), macrophage cluster 5 (Ccl8^+^ Cd209d^+^ macrophages) and macrophage cluster 6 (Ace^+^ Pglyrp1^+^ macrophages) (Figure 2A & B).

Interestingly, we found that macrophage clusters 0-6 were present at low levels in the Vsir^-/-^ psoriatic mice. Clusters 7 and 8 have very few cells and these clusters are contaminants of other cell types (Figure 2A). Cluster 1 expressed high levels of Ifit1, Rsad2, Cxcl10 and Oasl1. Besides, Cluster 2 expressed high levels of Slamf9, Lst1 and Tmem176a. It should be noted that major histocompatibility complex (MHC) class II molecule-related genes were expressed at high levels in cluster 3, such as H2-Eb1, H2-Aa and H2-Ab1. Cluster 4 expressed high levels of Ly6c2, Vcan and Chil3 (chitinase-like 3) 31, suggesting that these macrophages may be derived from monocytes (Figure S5). Clusters 0/1/2/3/4/5/6 expressed F13a1 and Ccl2. M1-associated genes (Il1b, Tnf, Cxcl10 and Ccl2) and M2-associated genes (Mrc1, encoding the mannose receptor, CD206) were expressed in cluster 1 (Figure 2C). H2-Eb1 was expressed in clusters 1 and 3. Psoriasis-like gene S100a4 32 was differentially expressed in clusters 4, cluster 3 and 6 (Figure S6).

The tSNE analysis was instrumental in revealing the heterogeneity between various macrophage clusters; however, it is possible that the clusters share common differentiation trajectories. Ordering of cells in pseudotime arranged the majority of macrophages into a major trajectory with two minor bifurcations. Macrophages from different subclusters were broadly distributed across the pseudotime space with macrophage cluster 2, 3 and 4 cells primarily occupying the left half of the major trajectory and the top right corner consisting of the cluster 1 cells. Clusters 0 and 5 were distributed preferentially at the minor bifurcation of the bottom right corner (Figure S6B). Macrophage cluster 4 expressed monocyte-like genes and located on the early time of the trajectory. Some of the macrophages in Vsir^-/-^ psoriatic mice were located on the minor bifurcation of the top right corner while additional macrophages were located on the left half of the major trajectory in WT psoriatic mice.

**Figure 3:**
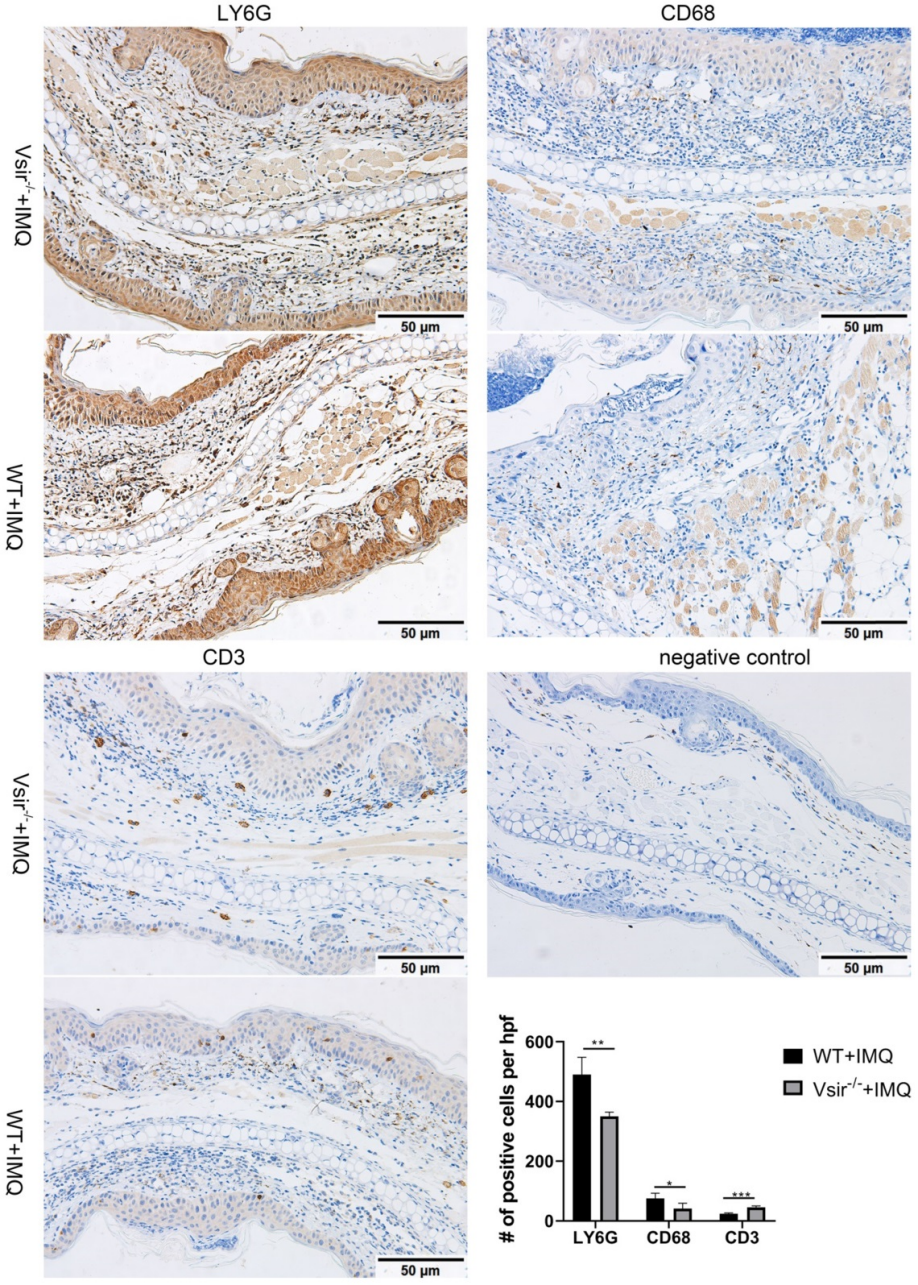
Immunohistochemistry of right ear skin lesion biopsies from WT and Vsir^-/-^ psoriatic mice show the expression of CD68, LY6G and CD3. Quantification of IHC staining from WT and Vsir^-/-^ psoriatic mouse skin lesions (n = 4) displayed as average number of positive cells per high-powered field (×200). Data are shown as the mean ± SEM. *P < 0.05, **P < 0.01, and ***P <0.001.

To determine whether the products of the genes are enriched in the macrophage subsets in murine psoriasis lesions, we performed immunohistochemical (IHC) analyses of skin biopsies and detected the products of macrophage-associated genes Cd68 and Ly6g enriched in the macrophages. We also detected the product of the Cd3e gene enriched in T cells. The result showed that CD68 and LY6G staining was more robust than the staining of CD3 in WT and Vsir^-/-^ psoriatic mouse skin lesions (Figure 3). The psoriasis skin lesions were infiltrated by macrophages. Hence, We found that macrophages rather than T cells played the dominant role in murine psoriasis. Moreover, CD68 and LY6G staining in WT psoriatic mice was stronger than that in Vsir^-/-^ psoriatic mice. Moreover, CD3 staining in Vsir^-/-^ psoriatic mouse skin lesions was stronger than that in WT psoriatic mice lesions (Figure 3). Thus, the IHC results are consistent with the results of the sc-RNA sequencing data.

### Transcriptional profile of skin myeloid dendritic cells/dendritic cell subsets was described

**Figure 4:**
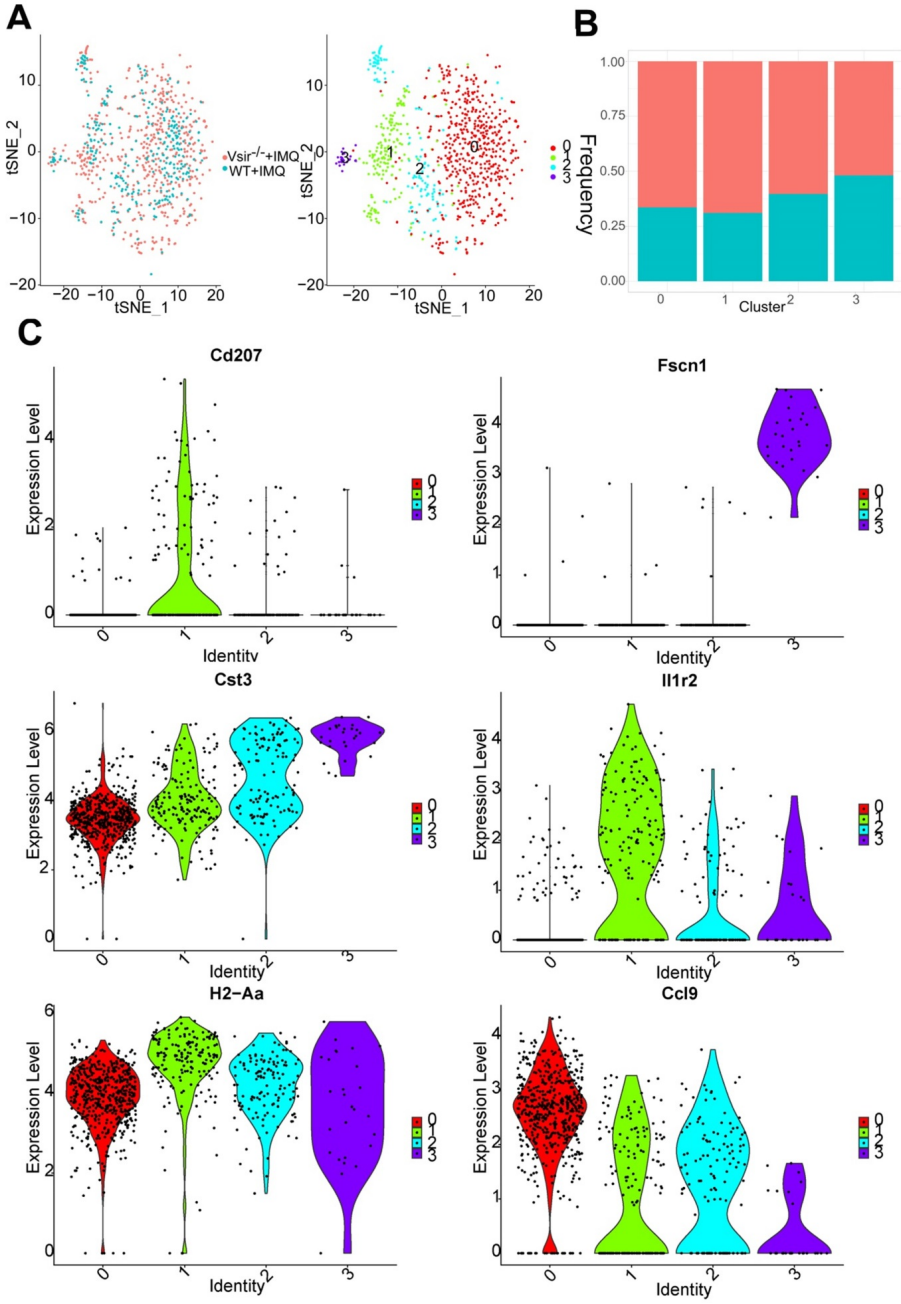
** DCs clusters were defined.** (**A**) The sample origin (left panel) and the tSNE plots of 868 DCs (right panel). (**B**) Bar plots show the cellular sources of DCs subtypes. Blocks represent different subjects and are colour-coded according to their derived groups defined in figure A. (**C**) Violin plots of the specific marker genes in each DC cell cluster. DCs, dendritic cells.

We detected a total of 868 DCs that formed 4 clusters. Indeed, DCs were expanded in Vsir^-/-^ psoriatic mice compared to WT psoriatic mice (Figure S4B). This cell population can be decomposed into DC cluster 0, DC cluster 1, DC cluster 2 and cluster 3 and various clusters expressed different marker genes (Figure S7A). DC cluster 0 (Fn1^+^ Lyz1^+^) was the major DC population in WT and Vsir^-/-^ psoriatic mice (Figure 4A). The percentage of DC cluster 1 (Cd207^+^ Il1r2^+^ DCs) and 2 (Clec9a^+^ Sept3^+^ DCs) was increased in Vsir^-/-^ psoriatic mice. DC cluster 3 appeared to be a steady DC cluster in both groups and was characterized by expression of DC maturation markers Fscn1 (Fascin1) 33 (Figure 4B & C). Interestingly, MHC class II molecules (H2-Aa) were highly expressed in four DC clusters (Figure 4C). In addition, Cd207 was a unique gene in cluster 1 and Il1r2 was expressed in all DC clusters except for DC cluster 0. Cd207 was a major marker of dermal DCs 34. Cst3 and certain chemokines Ccl9 were expressed in all DC clusters, Cst3 was expressed at high level in cluster 3 and Ccl9 were expressed at high levels in cluster 0.

We also investigate the heterogeneity of the DCs at a single-cell level. Using the machine-learning reverse graph embedding for dimensional reduction available in the Monocle 2 algorithm, we constructed a manifold using the DC cluster 0, 1, 2 and 3. This technique orders the single cells by expression patterns to represent distinct cellular fates or biological processes. We identified 5 states based on the changes in DCs transcriptomes. DC cluster 0 was predominantly distributed to state 3 and 4 and clusters 1 and 3 were placed on the late time of the trajectory (Figure S7B). Additional DCs in Vsir^-/-^ psoriatic mice were located on the late time of the trajectory while additional DCs were placed on the early time of the trajectory in WT psoriatic mice.

### Differential gene expression profiles of macrophages and dendritic cells in Vsir^-/-^ psoriatic mouse skin vs WT psoriatic mouse skin were revealed

**Figure 5:**
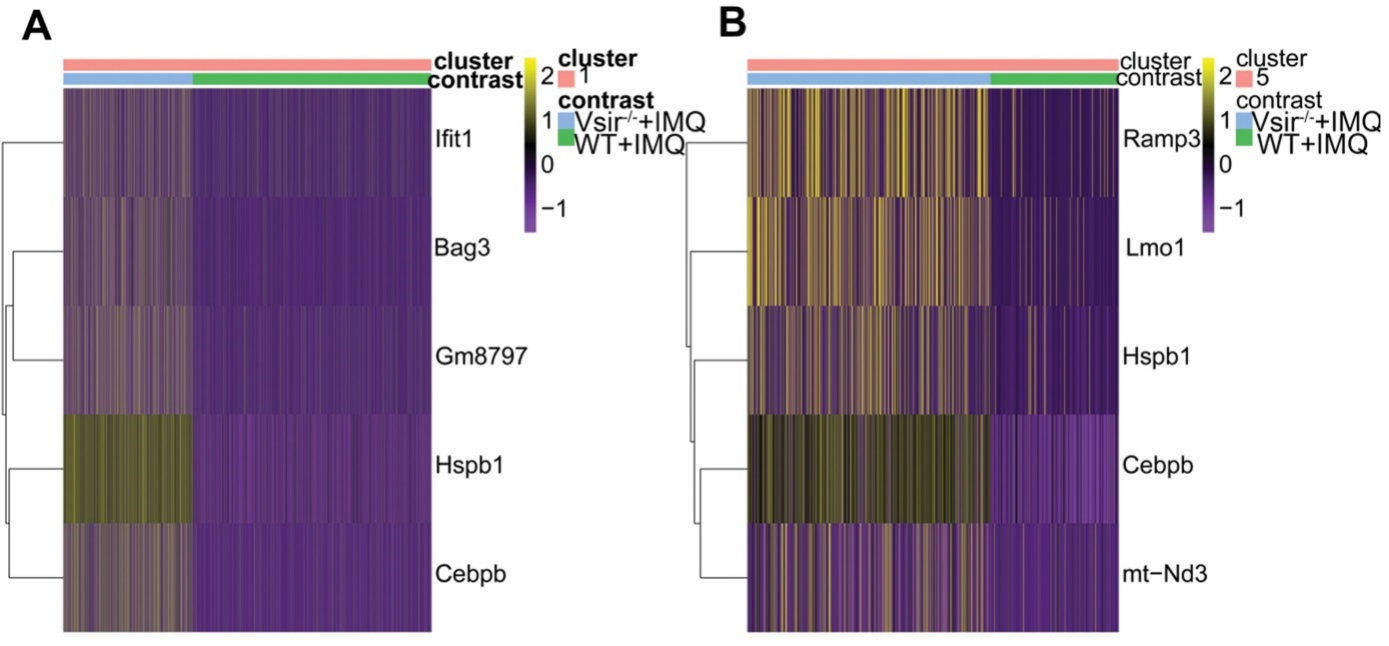
** Differential gene expression profile of macrophages and DCs in Vsir^-/-^ psoriatic mice vs WT psoriatic mice skin was analyzed.** (**A-B**) Heatmap of differentially expressed genes that were at least two-fold upregulated in macrophages (A) and DCs (B) from Vsir^-/-^ psoriatic mice. DCs, dendritic cells.

**Figure 6:**
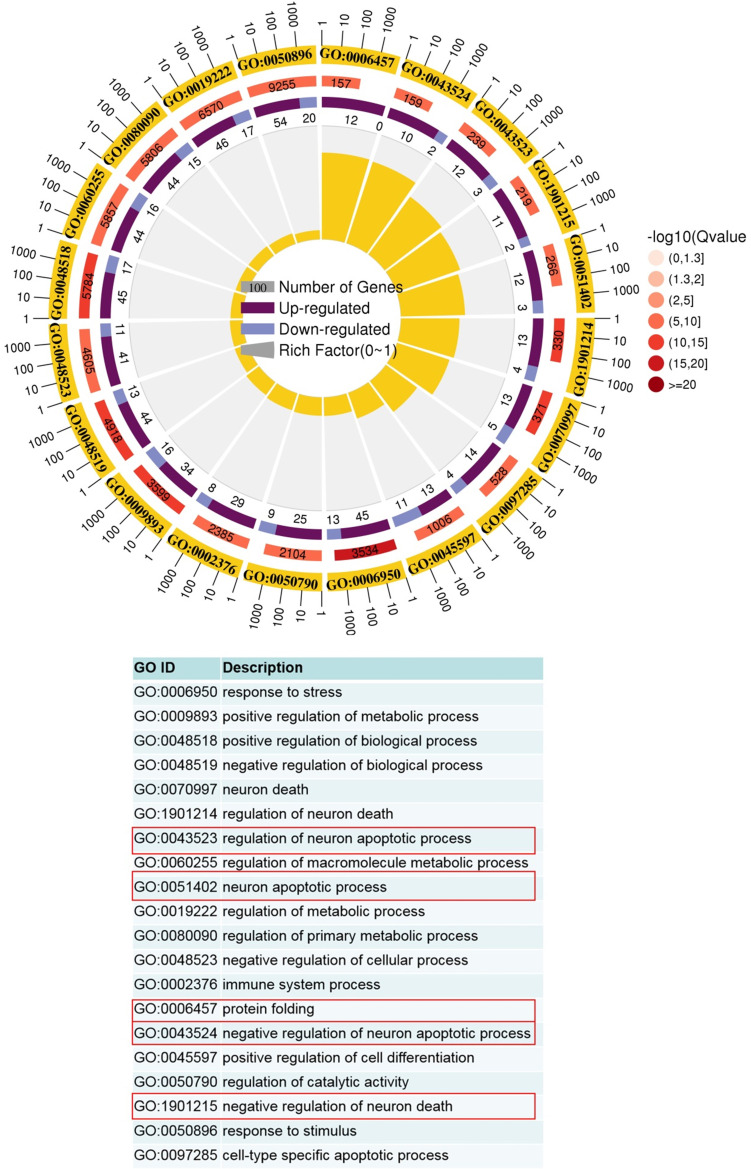
** GO term analysis of upregulated pathways in Vsir^-/-^ versus WT macrophages was performed.** The first lap indicates top 20 GO term and the number of the genes corresponds to the outer lap. The second lap indicates the number of the genes in the genome background and Q values for enrichment of the upregulated genes for the specified biological process. The third lap indicates the ratio of the upregulated genes (deep purple) and downregulated genes (light purple). The fourth lap indicates the enrichment factor of each GO term. GO, gene ontology.

After defining the macrophages in our dataset, we identified differentially expressed genes in WT versus Vsir^-/-^ psoriatic mouse skin lesions. We focused on the two-fold upregulated or two-fold downregulated genes in Vsir^-/-^ psoriatic mice macrophages compared to the levels in WT psoriatic mice macrophages. Heatmap for the expression of Hspb1 (heat shock protein 1), Cebpb (CCAAT/enhancer binding protein (C/EBP), beta), Gm8797 (ubiquitin B pseudogene), Bag3 (BCL2-associated athanogene 3) and interferon-stimulated genes Ifit1 (interferon-induced protein with tetratricopeptide repeats 1) demonstrated that these genes were upregulated in the macrophages from Vsir^-/-^ psoriatic mice lesions compared to the levels detected in WT psoriatic mice lesions (Figure 5A). Ifit1 was identified and validated as an important innate immune bottleneck 35. Hspb1 expression increased autophagic flux and inhibited apoptosis in renal tubular cells, indicating that Hspb1 upregulation plays a role in autophagy and apoptosis 36. Compared to the macrophages isolated from WT psoriatic mice, macrophages isolated from Vsir^-/-^ psoriatic mice have contributed to protein folding and negative regulation of the apoptotic process (Figure 6).

After defining the DCs in our dataset, we identified differentially expressed genes between WT and Vsir^-/-^ psoriatic mouse skin samples. Heatmap for the expression of Ramp3, Lmo1, Hspb1, Cebpb and mt-Nd3 demonstrated that these genes were upregulated in DCs from Vsir^-/-^ psoriatic mouse skin lesions compared to the levels detected in WT psoriatic mouse skin lesions (Figure 5B). C/EBPβ directly binds to the Il23r gene promoter in dendritic cells and drives autoimmunity and is a key driver of autoimmune inflammation in EAE 37. GO enrichment profile analysis demonstrated that the majority of the upregulated genes in the DC subsets are related to cytokine production and cell activation (Figure S8).

### Fibroblasts from Vsir^-/-^ psoriatic mice skin were enriched with macrophage-associated genes and inflammatory response, wound healing

**Figure 7:**
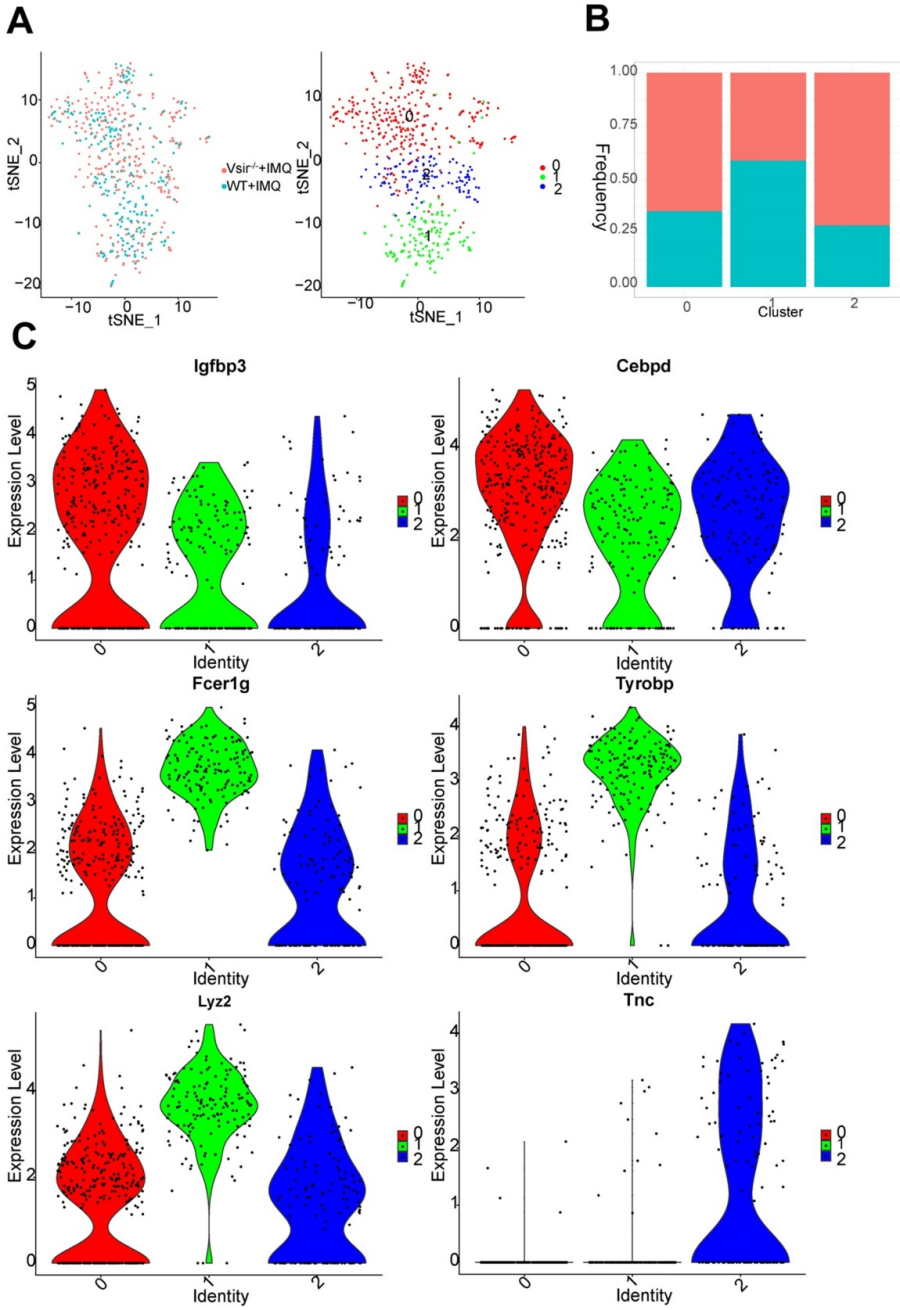
** Fibroblast cell clusters were described.** (**A**) The sample origin (left panel) and the tSNE plots of 561 fibroblast cells (right panel). (**B**) Bar plots corresponding to the cellular sources of the fibroblasts subtypes. Blocks represent different subjects and are colour-coded according to derived groups defined in figure A. (**C**) Violin plots of specific marker genes in each fibroblast cell cluster.

We detected 561 fibroblasts that were assembled into 3 clusters (Figure 7A). Fibroblasts were generally present at low levels in WT psoriatic mice (1.89%) but proliferated in Vsir^-/-^ psoriatic mice (4.13%) (Figure S4B). In all fibroblasts populations, the clusters 0 and 2 population was increased in Vsir^-/-^ psoriatic mice (Figure 7B). Fibroblast cluster 0 was characterized by Mgp and Fmo2 expression. Cluster 1 was characterized by Cxcl1 and Clec4b1 expression. Tnc and Chl1 were the marker genes of cluster 2, which also expressed Cyp26b1, Sectm1a and Cpxm1 (Figure S9A). Igfbp3 and Cebpd were expressed in all of the three fibroblast clusters. Macrophage-associated genes Fcer1g (Fc fragment of IgE receptor Ig) 38, Lyz2 (lysozyme 2) 39 and Tyrobp (TYRO protein tyrosine kinase binding protein) 38 were differentially expressed in cluster 1. Comparison of fibroblast clusters, we identified Tnc as unique gene in cluster 2 (Figure 7C).

We identified differentially expressed genes in fibroblasts in WT and Vsir^-/-^ psoriatic mouse skin. Heatmap for the expression of Ptx3, Hsbp1, Cebpb and Tnfrsf12a demonstrated that these genes were upregulated, while S100a9 was downregulated in the fibroblasts from Vsir^-/-^ psoriatic mouse skin compared to the levels detected WT psoriatic mouse skin (Figure S9B). We identified functional characteristics of the fibroblasts in various groups. The functions of the fibroblasts in Vsir^-/-^ psoriatic mice included inflammatory response, wound healing and response to wounding (Figure S10).

## Discussion

Detailed analysis of the role of VISTA in human psoriasis have not yet been performed; however, it should be noted that the human and murine VISTA proteins share 90% identity and display similar expression patterns 40. VISTA is an important immune checkpoint regulator in the maintenance of skin homeostasis and inflammation. The genetic deletion (e.g., PD-1, analogous to VISTA) in mice results in autoimmunity; VISTA fusion protein (VISTA.COMP) reduced acute inflammatory hepatitis in an experimental model 41. On the other hand, VISTA-deficient mice develop spontaneous autoimmunity resembling lupus 11. Moreover, Vsir^-/-^ mice developed exacerbated psoriasiform inflammation. The immune cells subsets and their roles in Vsir^-/-^ psoriatic mice are largely unknown. IMQ-treated Vsir^-/-^ mice had more severe ear swelling when compared to that in WT mice (Figure S1).

Advances in scRNA-seq technology enabled comprehensive analysis of the immune system in an unbiased way at a single cell level. To the best of our knowledge, the present study is the first comprehensive, high-resolution, single cell transcriptomic analysis of immune cell types and expression programmes that compares Vsir^-/-^ psoriatic mice with WT controls. Here, we provide a comprehensive analysis of scRNA-seq data generated from the cells derived from WT and Vsir^-/-^ psoriatic mice back skin. Our atlas of Vsir^-/-^ and WT psoriatic mouse skin comprises 12 broadly defined cell types. In our study, VISTA deficiency decreased macrophages populations and augmented the number of DCs, T cells, fibroblasts, NK cells, endothelial cells, mast cells, and B cells resulting in exacerbation of psoriasiform dermatitis.

Our scRNA-seq findings indicated that macrophages are the largest cell population in the two groups (more than 60%) (Figure S4B). The results of immunohistochemistry verified extensive macrophages infiltration in the mouse psoriasis lesions (Figure 3). Our results suggest that macrophages, as innate immune cells, play important roles in psoriasis. Interestingly, the macrophage population was decreased in the Vsir^-/-^ group. The expression of certain genes may have a function in Vsir^-/-^ psoriatic mice. We noted that the expression levels of Hspb1, Cebpb, Gm8797, Bag3 and interferon-stimulated gene Ifit1 were upregulated in the macrophages from Vsir^-/-^ psoriatic mice skin lesions compared to WT psoriatic mouse skin lesions (Figure 5A). The change in these genes may be related to the deterioration of skin inflammation in Vsir^-/-^ psoriatic mice. For example, previous studies 42 demonstrated that C/EBPβ-deficient alveolar macrophages released significantly less TNF-α and IL-6; moreover, mice carrying a targeted deletion of the C/EBPβ gene displayed significant attenuation of morphological lung injury. These observations may explain, at least in part, the exacerbated inflammatory phenotype observed in Vsir^-/-^ psoriatic mice. In addition, the mechanism of the induction of interferon-stimulated genes (ISG) induction in HIV-1-infected macrophages has been studied 43. Ifit3 has an antiviral function in macrophages and enhances immune response. Thus, VISTA may drive the pathology of psoriasis by increasing the upregulate of specific genes in the macrophages.

The Cebpb gene expression in the majority of the cell populations (e.g. macrophages, DCs, T cells and fibroblasts) was higher in Vsir^-/-^ psoriatic mice compared with that in WT psoriatic mice (Figure 5A, 5B, S14A and S9B). Cebpb (also known as: C/EBPβ) is an intronless gene encoding three protein isoform, LAP1, LAP2, and LIP 44. C/EBPβ induces a variety of genes that orchestrate immune responses, including cytokines, chemokines and their receptors. Historically, C/EBPβ has been extensively studied in the setting of IL-1 and lipopolysaccharide (LPS) signaling 45, 46. Previous studies have shown that C/EBPβ contributes to immunity to mucosal candidiasis during cortisone immunosuppression in a manner linked to β-defensin 3 expression 47. Thus, C/EBPβ plays an important role in inflammatory response. C/EBPβ itself regulates numerous genes involved in inflammation and plays an unexpected role in regulation of Il23r expression in antigen-presenting cells (APCs) 37. We have identified differentially expressed genes in immune cells of WT and Vsir^-/-^ psoriatic mouse skin, and found that Cebpb was upregulated in macrophages (Figure 5A), DCs (Figure 5B), T cells (Figure S14A), neutrophils (Figure S11D), fibroblasts (Figure S9B), endothelial cells (Figure S18B) and adipocytes (Figure S18A) of Vsir^-/-^ psoriatic mice. Thus, C/EBPβ may play an important role in Vsir^-/-^ psoriatic mice.

Hspb1 is another expressed gene with high expression levels in the majority of cellular subsets (e.g. macrophages, DCs, fibroblasts and NK cells) of Vsir^-/-^ psoriatic mice (Figure 5A, 5B, S9B and S18C). Small heat shock protein beta-1 (Hspb1) (also known as mouse HSP25 and human HSP27) is a prominent and well characterized member of the small HSP (sHSP) subfamily 48. A wide variety of physiological and environmental stimuli can induce expression of HSPs, including physical and biological insults (e.g., inflammation) 49. As molecular chaperones, HSPs play central roles in protein folding and cytoprotection by preventing post-mitochondrial apoptosis in the caspase-dependent pathways (e.g., Hspb1) 50. Previous observations suggest that the expression of Hsps in the neoepidermis is related to the proliferation, migration, and differentiation states of keratinocytes within the wound 51. The rate of wound healing was significantly impaired in Hspb1-deficient mice characterized by reduced re-epithelialisation 52. We identified differentially expressed genes in immune cells between WT and Vsir^-/-^ psoriatic mouse skin, and found that Hspb1 was upregulated in macrophages (Figure 5A), DCs (Figure 5B) and neutrophils (Figure S11D) of Vsir^-/-^ psoriatic mice. Thus, excessive proliferation of keratinocytes in Vsir^-/-^ psoriatic mice may be related to upregulation of the Hspb1 gene.

In this study, comparison of Vsir^-/-^ psoriatic mice with WT psoriatic mice indicated that macrophages isolated from Vsir^-/-^ psoriatic mice have contributed to the negative regulation of apoptosis (Figure 6). Neutrophils isolated from Vsir^-/-^ psoriatic mice may have contributed to negative regulation of apoptotic process, negative regulation of programmed cell death and positive regulation of immune system process (Figure S12). Hydrogen peroxide induces eosinophil apoptosis and promotes the resolution of allergic inflammation 53. Apoptosis of inflammatory cells may contribute to the resolution of psoriatic inflammation. Moreover, the majority of the upregulated genes in the DC subsets are related to cytokine production and cell activation (Figure S8). The changes may contribute to aggravation of skin inflammation in Vsir^-/-^ psoriatic mice.

Previous studies in Vsir^-/-^ psoriatic mice suggested that VISTA is a critical regulator of the inflammatory responses mediated by DCs. In the present study, an increase in DCs in Vsir^-/-^ psoriatic mice was verified. Previous findings highlighted the potent autostimulatory potential of psoriatic plaque skin-derived DCs and suggested an important immunological contribution of cell types contained within the skin lesions 54. DCs may be an important in earliest indicator of psoriasis; psoriasis lesion-derived DCs stimulated a T cell response with production of IL-2 and IFN-γ 55. Our scRNA-seq findings demonstrated that DCs were increased in Vsir^-/-^ psoriatic mice compared with that in WT psoriatic mice (Figure S4B); hence, the deletion of VISTA may function during the early stages of psoriasis.

Numerous studies have demonstrated that psoriasis lesions contain increased numbers of T cells 56. A total of 625 T cells were detected (Figure S13). Approximately 5.82% of skin cells were T cells in Vsir^-/-^ psoriatic mice, and only 1.27% skin cells were T cells in WT psoriatic mice (Figure S4B). Since T cells occupied a small population compared with other immune cells here, we presume that T cells may not play as important role as macrophages in Vsir^-/-^ psoriatic mice. To further demonstrate the observable differences in the T cells, we performed an integrative analysis of the T cell receptor (TCR) repertoire. We compared the clonotypes (TCR alpha and beta chains) found in the WT and Vsir^-/-^ psoriatic mice with the TCR databases. To adequately define T cell clonality, we strictly defined T cells with at least one pair of identical paired α-β chains to be a single clone from the same ancestry, and the expanded clones were defined as those whose α and β TCR pairs were shared by at least three cells in a given cell population. We noted that the majority of the cells contained unique TCRs in both groups of mice. Only~10% T cells harboured clonal TCRs in WT psoriatic mice; however, Vsir^-/-^ psoriatic mice had a substantially higher percentage at~20% (Figure S14B). Thus, single cell V-D-J sequencing analysis did not reveal significant differences in the abundance or cell state of various T cell subsets between the WT and Vsir^-/-^ psoriatic mice (Figure S14B).

We identified a number of candidate genes preferentially enriched in immune populations; harnessing these genes may enable subsequent exploration and targeting of distinct macrophage populations and their functions in psoriasis, providing a valuable resource in the field. The causal role of macrophages in various inflammatory diseases is only beginning. Therefore, exacerbated psoriasiform inflammation in Vsir^-/-^ mice may be related to the Hspb1 and Cebpb genes.

In conclusion, based on an unbiased single-cell RNA-seq approach, we established the transcriptional signature of major immune cell populations and identified their gene expression signature, suggesting specialized functions in Vsir^-/-^ murine psoriasis. Our findings offer an insight into the function of VISTA in psoriasis and provide a compilation of gene expression signatures for subsequent detailed investigations of the complexities of skin that will serve as the foundation for future studies of psoriasis.

## Supplementary Material

Supplementary figures and tables.

## Abbreviations

APC: antigen presenting cells
Bag3: bcl2-associated athanogene 3
Cebpb: CCAAT/enhancer binding protein (C/EBP), beta
Chil3: chitinase-like 3
CNS: central nervous system
DCs: dendritic cells
EAE: experimental autoimmune encephalomyelitis
EC: endothelial cells
Gm8797: ubiquitin B pseudogene
GO: gene ontology
H&E: hematoxylin-eosin
Hspb1: heat shock protein 1
Ifit1: interferon-induced protein with tetratricopeptide repeats 1
IHC: immunohistochemistry
IMQ: imiquimod
ISG: interferon-stimulated genes
LPS: lipopolysaccharide
MAST: model-based analysis of single-cell transcriptomics
MHC: major histocompatibility complex
NK cells: natural killer cells
PCA: principal component analysis
scRNA-seq: single-cell RNA sequencing
TCR: T cell receptor
TLR7: toll-like receptor 7
tSNE: t-distributed stochastic neighbour embedding
UMIs: unique molecular identifiers
VISTA: V-domain immunoglobulin suppressor of T cell activation
Vsir^-/-^: Vsir knockout mice
WT: wild type
