# Supplementary material for: Single-cell RNA-Seq reveals the transcriptional landscape and heterogeneity of skin macrophages in Vsir-/- murine psoriasis
Source: Theranostics. 2020 Aug 21;10(23):10483–97. doi: 10.7150/thno.45614 (PMC7482809; doi:10.7150/thno.45614)
Supplement: Supplementary file 1 — Supplementary figures and tables. [file thnov10p10483s1.pdf]

Supplemental Results

Transcriptional profile of neutrophil cell subsets was described in murine psoriasis.

A total of 1,261 neutrophil cells that were clustered into 5 clusters (Figure S11A). Neutrophils constituted 5.91% of total cells in Vsir$^{-/-}$ psoriatic mice. Most interestingly, this percentage was a little lower than that in WT psoriatic mice (6.56%) (Figure S4B). We noted that, while neutrophil cluster 0 (Ube2c^{+} Rrm2^{+}) and 1 (Apoe^{+} C4b^{+}) were the main neutrophil clusters involved in both groups, cluster 3 (Lcn2^{+} Ngp^{+}) was present at high level in Vsir$^{-/-}$ psoriatic mice (Figure S11A-B). Flt3 and Clec9a were marker genes of cluster 4, which also expressed Klrd1, H2-Ob and Sept3. Cluster 2 expressed Chil3, Vcan and Plac8 (Figure S11C).

After defining the neutrophils in our dataset, we identified differentially expressed genes between WT and Vsir$^{-/-}$ psoriatic mice of the skin. We focused on the two-fold upregulated or two-fold downregulated genes in neutrophils of Vsir$^{-/-}$ psoriatic mice neutrophils compared to that of WT psoriatic mice. Heatmap for the expression of Hspb1, Cebpb, Gm8797 and Lyz1 demonstrated that these genes were upregulated, while Stab1 was downregulated in DCs from Vsir$^{-/-}$ psoriatic mice lesions compared to WT psoriatic mice lesions (Figure S11D). Compared to those isolated from WT psoriatic mice, neutrophils isolated from psoriatic Vsir$^{-/-}$ mice may have contributed to negative regulation of apoptotic process, negative regulation of programmed cell death and positive regulation of immune system process (Figure S12).

T cell cluster was identified in Vsir$^{-/-}$ psoriatic mice.

A total of 625 T cells were detected (Figure S13A). Cd3e and Cxcr6 were marker genes of T cells, which also expressed Trdv4, Cd3d and Cd3g (Figure S13B). T cells were generally present at low levels in WT psoriatic mice but proliferated in Vsir$^{-/-}$ psoriatic mice (Figure S4B). But approximately 5.82% of skin cells were T cells in Vsir$^{-/-}$ psoriatic mice, only 1.27% skin cells were T cells in WT psoriatic mice (Figure S4B). Based on these results, We suspected that T cells may not play major role such as macrophages in psoriatic mice.

After defining the T cells, we identified differentially expressed genes between the skin lesions of WT and Vsir$^{-/-}$ psoriatic mice. We focused on the two-fold upregulated or two-fold downregulated genes in T cells of Vsir$^{-/-}$ psoriatic mice T cells compared to that of WT psoriatic mice. Heatmap for the expression of Crem, Cebpb, Gzma and Tnfrsf9 demonstrated that these genes were upregulated, while Plaur was downregulated in T cells from Vsir$^{-/-}$ psoriatic mouse skin compared to WT psoriatic mouse skin lesions (Figure S14A). Furthermore, T cells isolated on Vsir$^{-/-}$ psoriatic mice were shown to be involved in regulation of leukocyte differentiation, regulation of hemopoiesis and leukocyte cell-cell adhesion (Figure S15).

We defined clonotypes based on CDR3 sequences of both TCR α and β chains using the Cell Ranger analysis pipeline. To adequately define T cell clonality, we strictly defined T cells with at least one pair of identical paired α-β chains to be one clone from the same ancestry, and the expanded clones were defined as those whose α and β TCR pairs were shared by at least three cells in a given cell population. We noted that, most cells contained unique TCRs in both two groups of mice. While only~10% T

cells harbored clonal TCRs in WT psoriatic mice, Vsir$^{-/-}$ psoriatic mice showed a much higher percentage at~20% (Figure S14B).

Other small cell clusters were detected in murine psoriasis.

In addition to the large populations of immune cells detected during murine psoriasis described above, other cell types were separated into 7 clusters, including natural killer (NK) cells, epithelial cells, endothelial cells, mast cells, B cells, myocytes and adipocytes (Figure S16). In addition, the different clusters were characterized by the expression of other relevant markers (Figure S17). We identified the NK cells characterized by Ncr1, Klra4, Klra8 and Gzma expression. Ifng also highly expressed in NK cells (Figure S17A). The proportion of NK cells increased in psoriatic Vsir$^{-/-}$ mice (Figure S4B). And NK cells played roles in the protein refolding in psoriatic Vsir$^{-/-}$ mice (Figure S19). Epithelial cells were identified by expression of Defb6, Krt15, Cst6 and Pla2g2f (Figure S17B). Gene expression patterns of established canonical markers of endothelial cells (Emcn, Sox17, Cldn5, Esam), mast cells (Fcer1a, Tpsg1, Slc6a4, Cyp11a1) and B cells (Iglv1, Jchain, Cd79a, Iglc2) allowed us to assign putative biological identities to these clusters (Figure S17). Furthermore, these three clusters increased in psoriatic Vsir$^{-/-}$ mice (Figure S4B). The increase of endothelial cells in Vsir$^{-/-}$ psoriatic mice may be relative to angiogenesis. B cells, epithelial cells and adipocytes contained the large numbers of UMIs. This finding might suggest that these clusters were more "active" (Figure 1C).

Supplementary Figures

Figure S1.

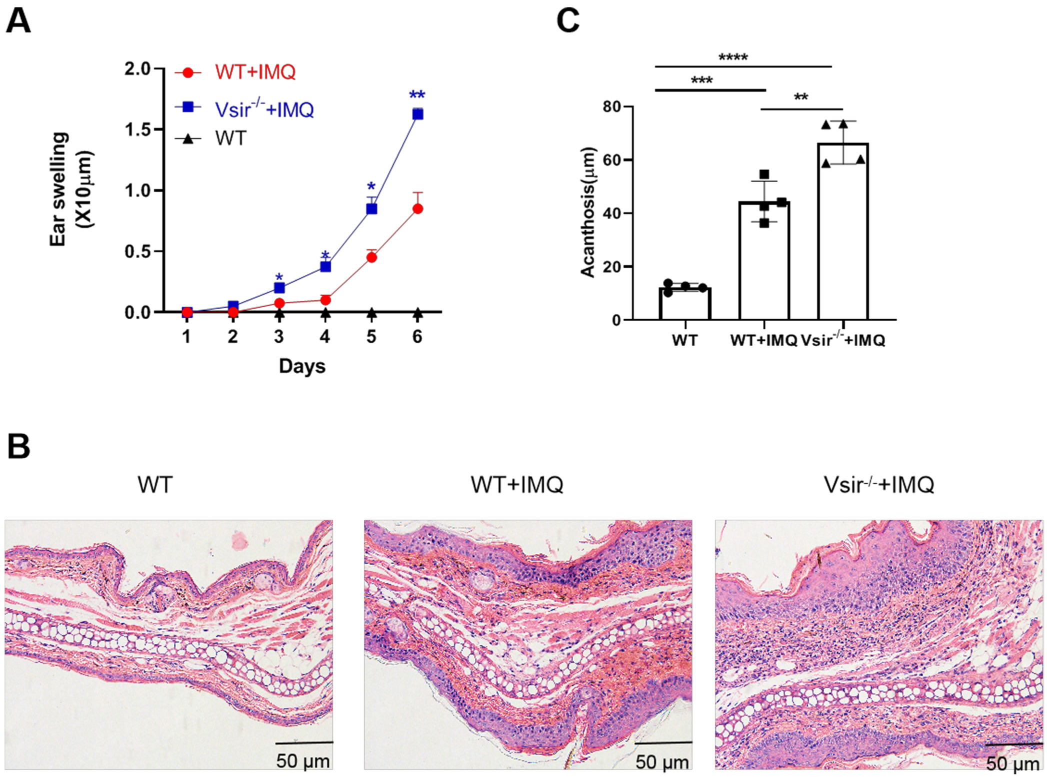

Figure S1. VISTA deficiency exacerbated the IMQ-induced psoriasiform inflammation.

(A) WT and $Vsir^{-/-}$ mice were topically treated daily on right ear and back skin with 5% IMQ cream for 5 days. Ear thickness was measured daily. Ear swelling is shown as the increase of ear thickness when compared to day 0, and expressed as mean ± SEM (n = 4). **(B)** H&E staining of the right ear skin of WT+IMQ and $Vsir^{-/-}$+IMQ mice; Scale bar 50 μm. (×200). **(C)** Ear skins were collected for H&E staining and analyzed for average acanthosis of ear. n=4. H&E, hematoxylin-eosin. IMQ, imiquimod. $Vsir^{-/-}$, Vsir knockout mice. WT, wild type.

Figure S2.

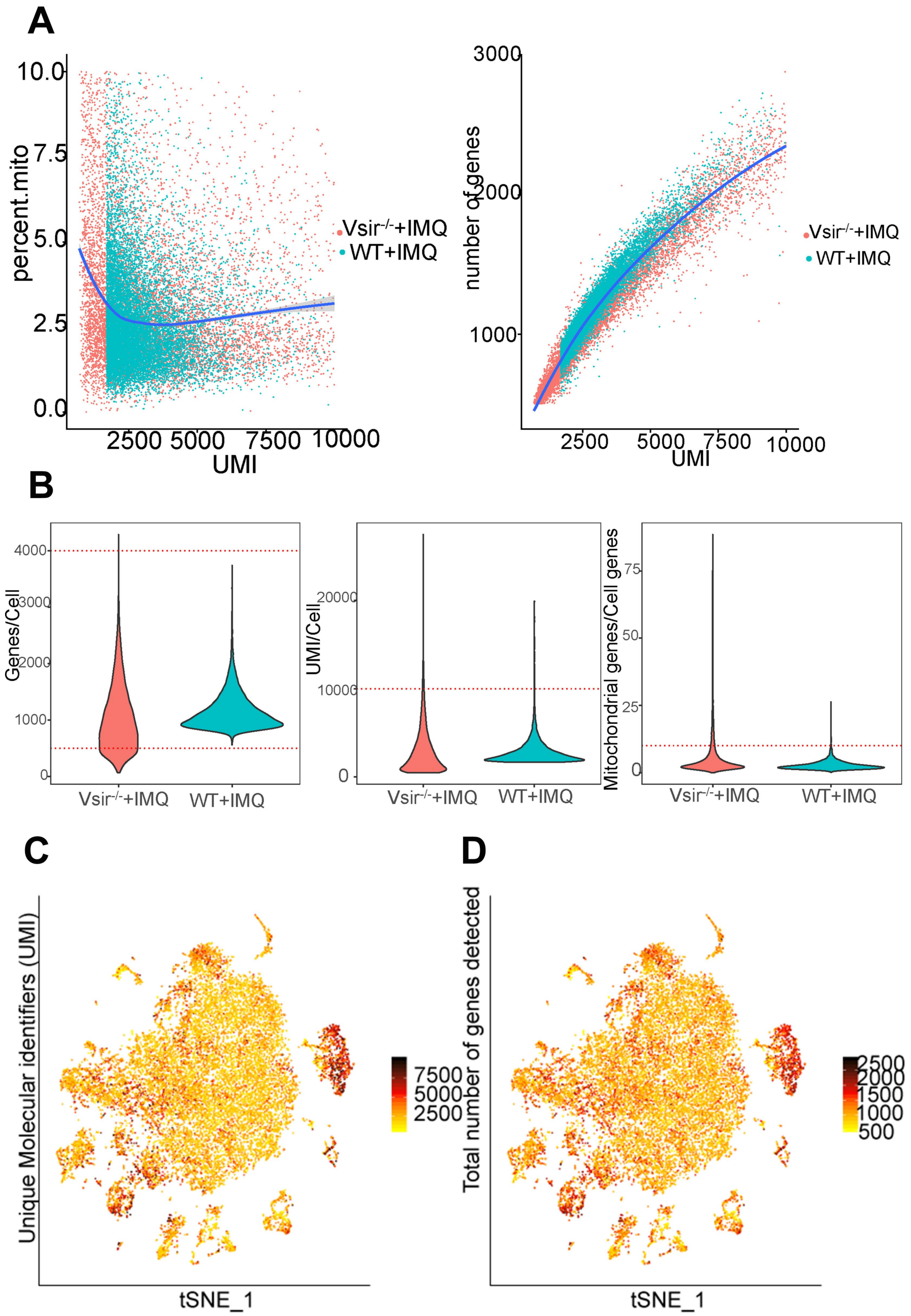

Figure S2. Quality control metrics of scRNA-seq data from Vsir$^{-/-}$ psoriatic mice

were analyzed.

(A) The percent of mitochondrial genes were shown. **(B)** Genes/Cell, Unique Molecular Identifiers (UMI)/Cell, and percent of mitochondrial genes/cell genes were shown. **(C)** tSNE plot with color-coded UMIs per cell is shown. Cells with the highest UMI are colored black. **(D)** tSNE plot with color-coded number of expressed genes per cell was shown. Cells with the highest number of expressed genes are colored black. tSNE, t-distributed stochastic neighbour embedding. UMIs, unique molecular identifiers.

Figure S3.

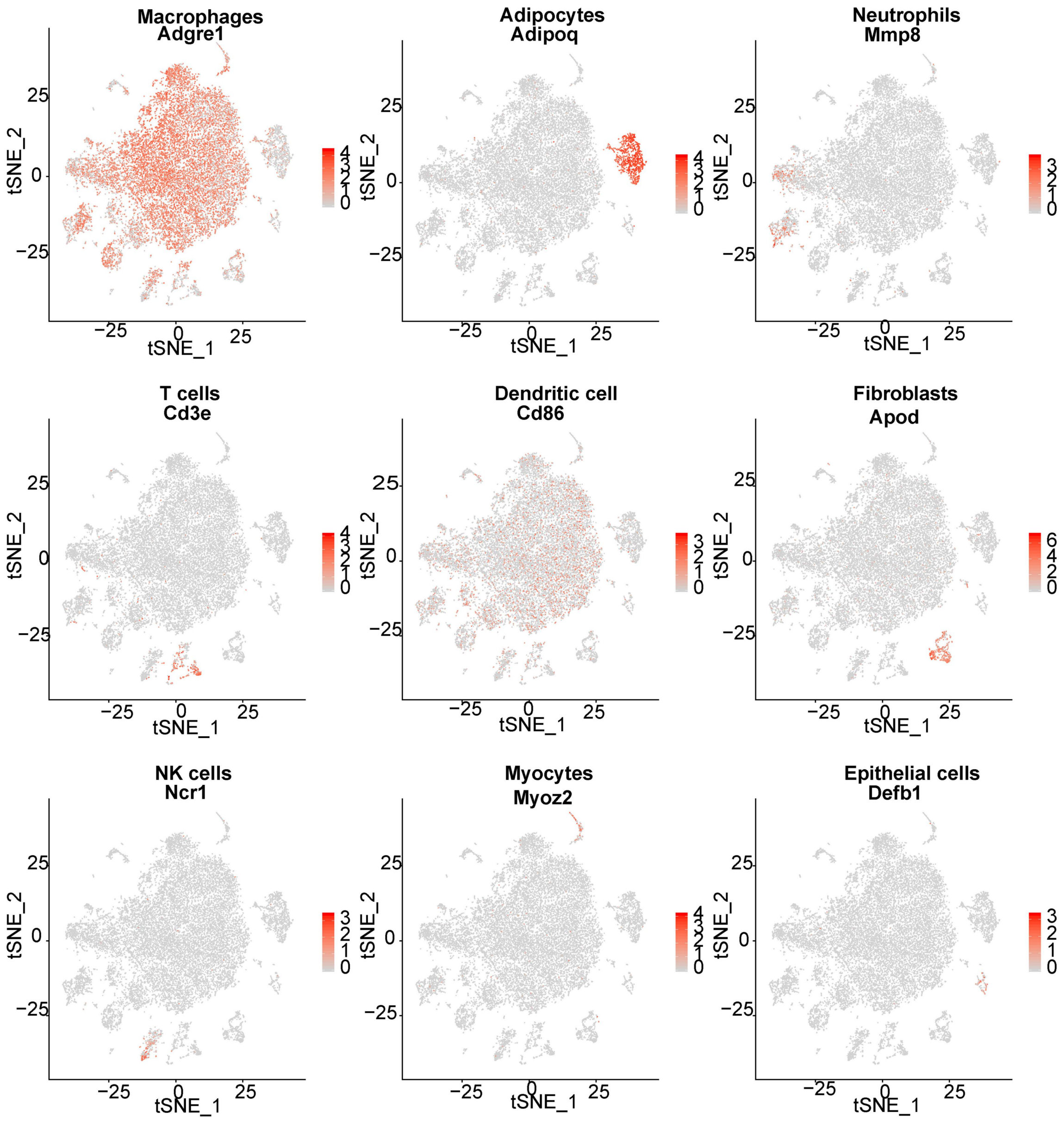

Figure S3. Cell types were identified.

Expression of the classical marker genes used to define different cell types. NK cells, natural killer cells.

Figure S4.

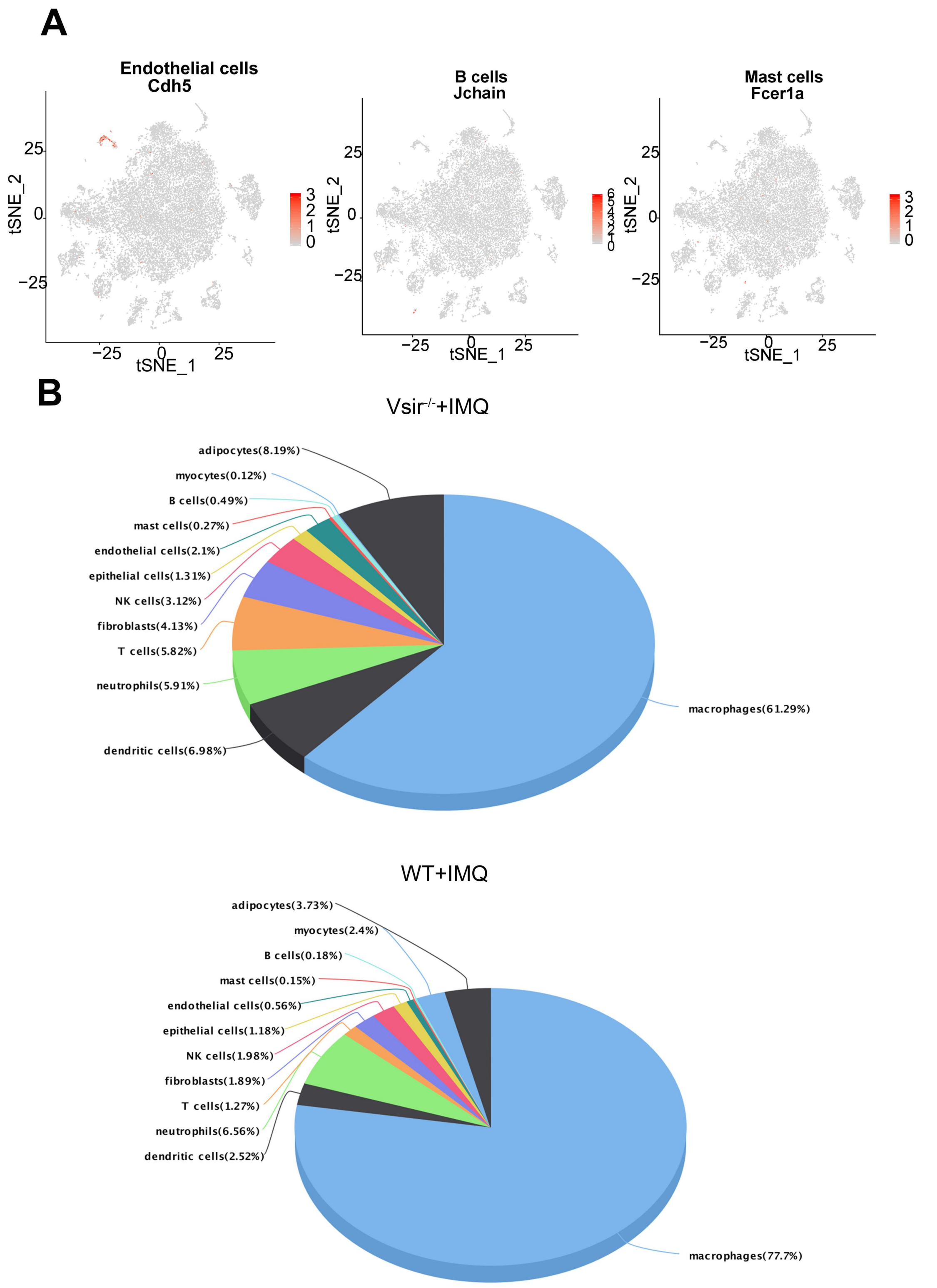

Figure S4. Proportions of all cell populations in WT and Vsir$^{-/-}$ psoriatic mice

were shown.

(A) Expression of the classical marker genes used to define different cell types. **(B)** Proportions of all cell populations within total skin cells in WT and $Vsir^{-/-}$ psoriatic mice.

Figure S5.

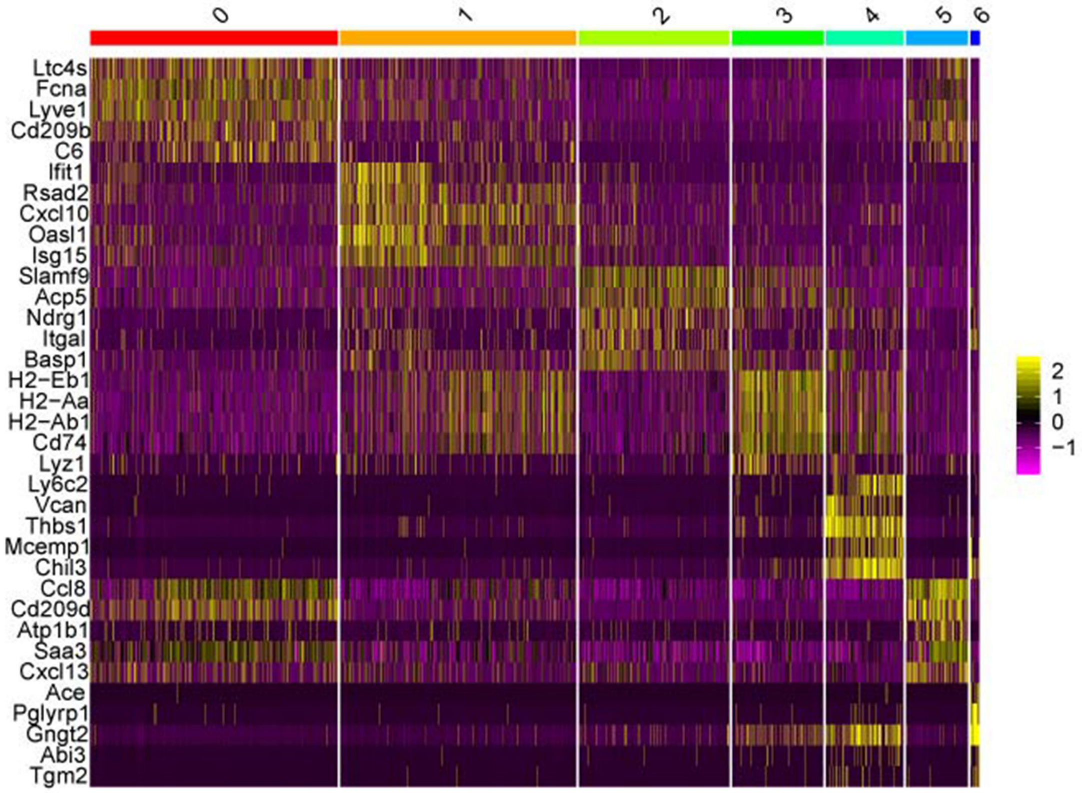

Figure S5. Specific marker genes in each macrophage cluster were show in heatmap.

Figure S6.

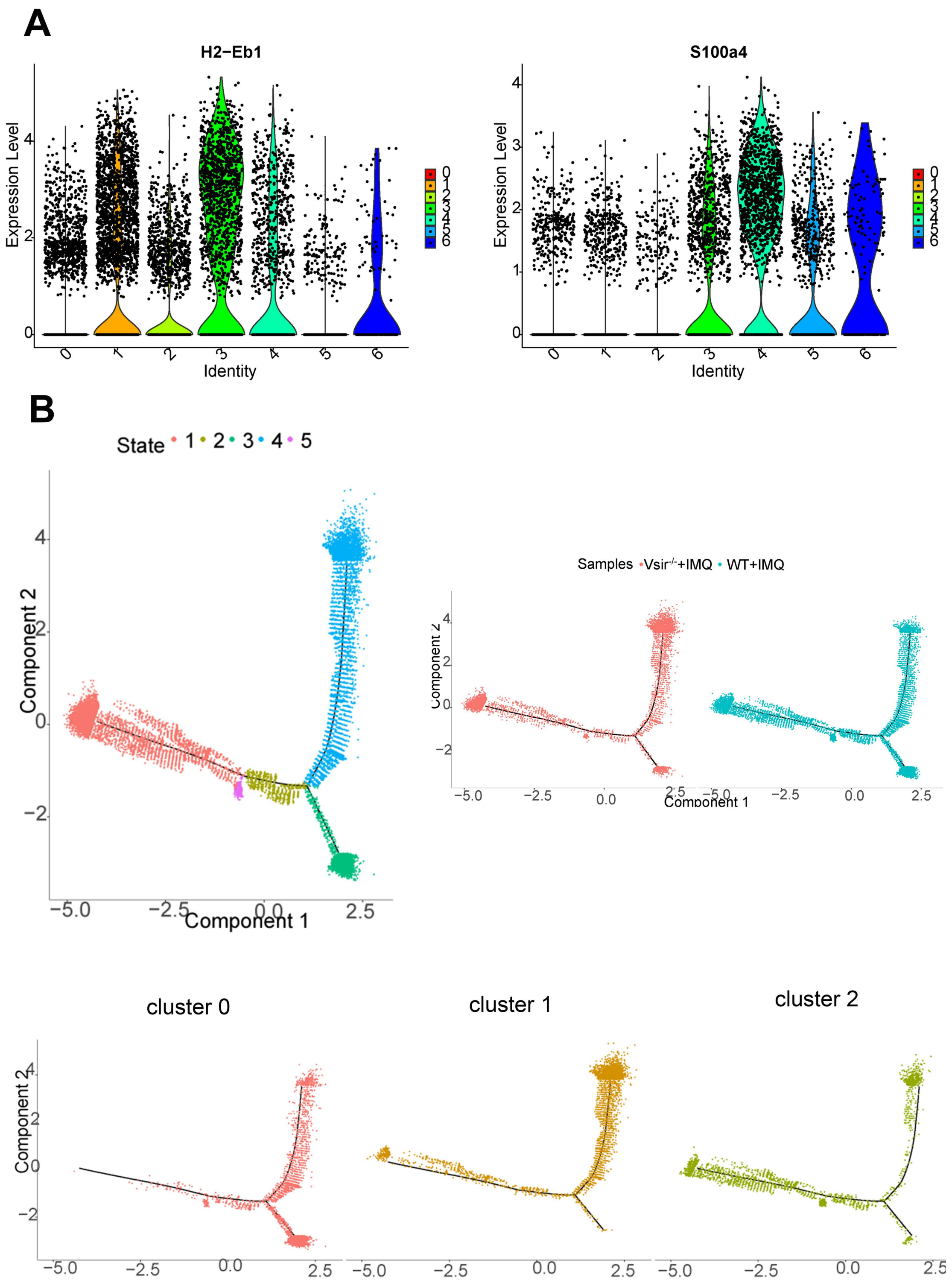

Figure S6. Signature genes and distinct cell trajectory states for macrophage cell clusters were described.

(A) Violin plots of specific marker genes in each macrophage cluster. **(B)** Distinct cell trajectory states defined by single cell transcriptomes and pseudotime trajectory of macrophages shown separately for WT and $Vsir^{-/-}$ psoriatic mice (top panel). Trajectory of macrophages from clusters 0, 1 and 2 using the Monocle 2 algorithm (bottom panel).

Figure S7.

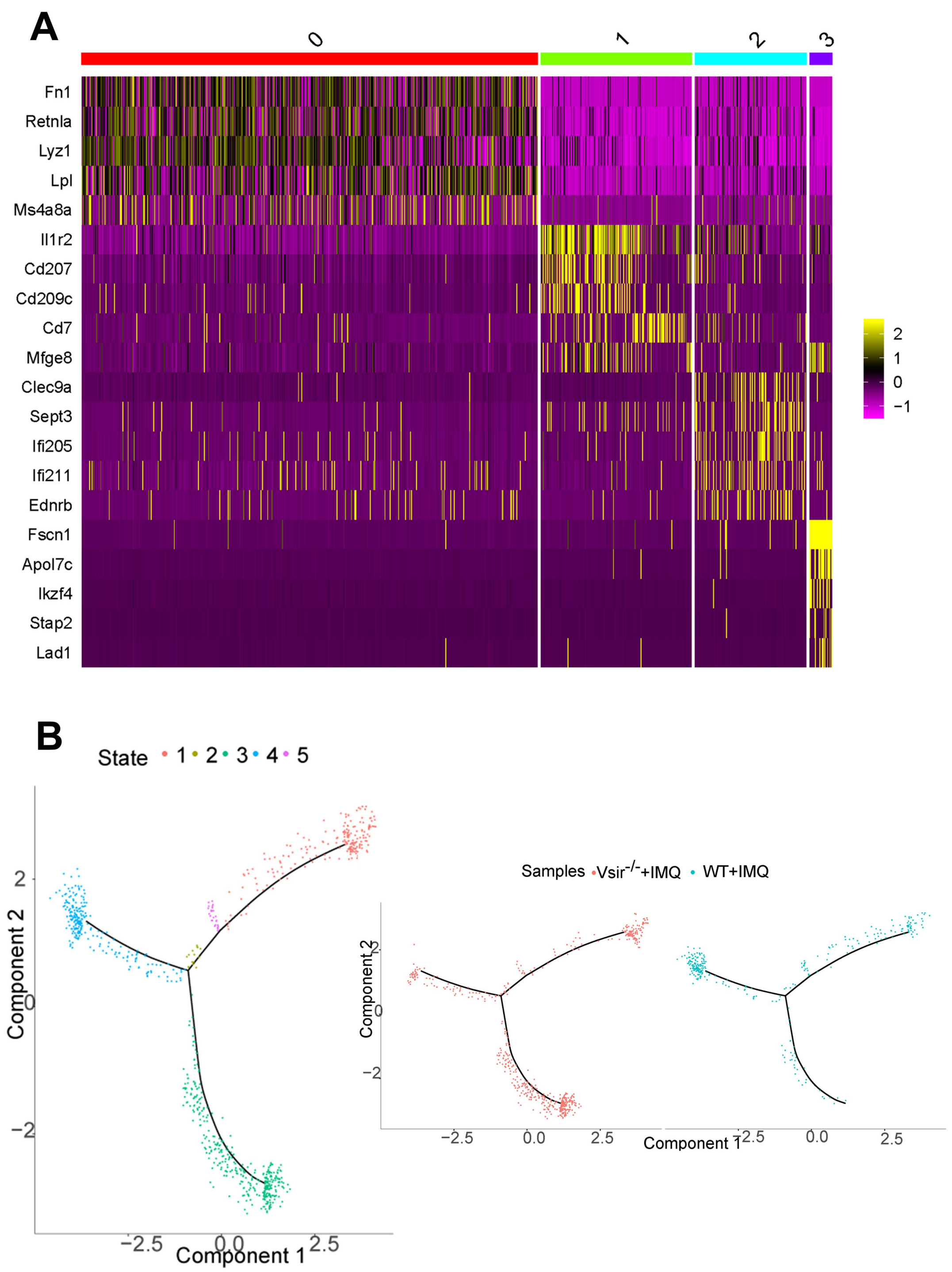

Figure S7. Signature genes and distinct cell trajectory states for DC clusters were described.

(A) Heatmap showing specific marker genes in each DC cluster. **(B)** Trajectory of DCs from clusters 0, 1, 2 and 3 using the Monocle 2 algorithm. DCs: dendritic cells.

Figure S8.

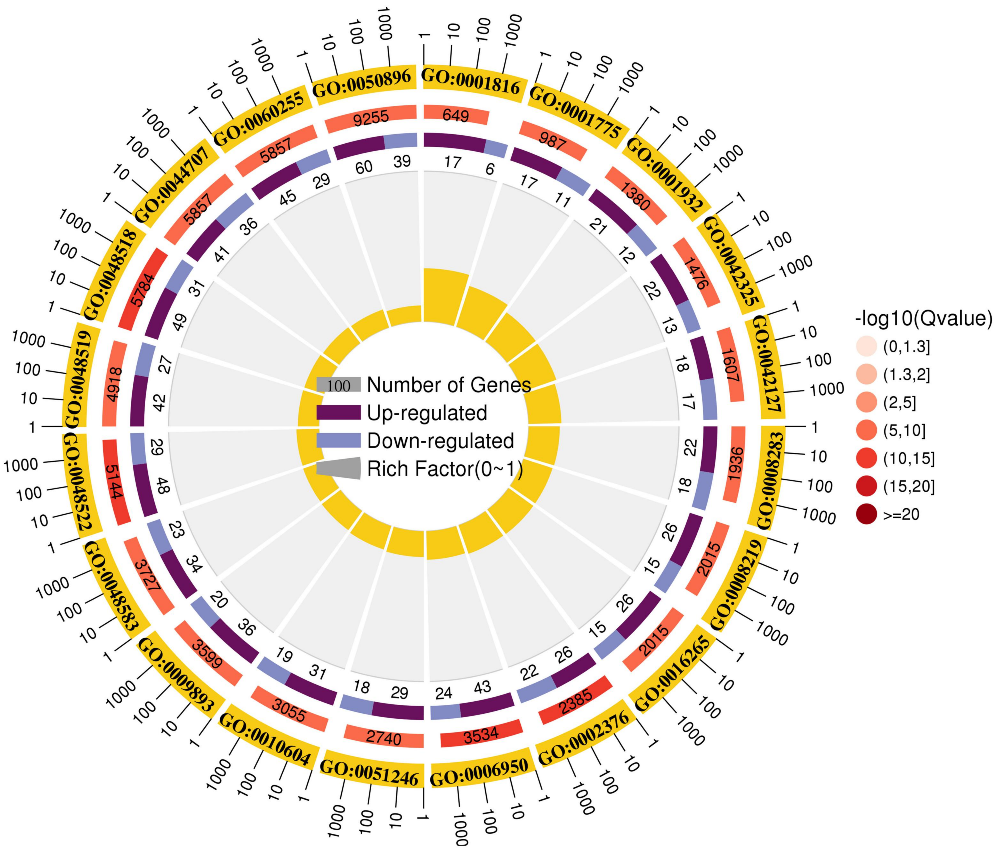

GO ID	Description
GO:0006950	response to stress
GO:0048522	positive regulation of cellular process
GO:0048518	positive regulation of biological process
GO:0002376	immune system process
GO:0044707	single-multicellular organism process
GO:0048519	negative regulation of biological process
GO:0050896	response to stimulus
GO:0042325	regulation of phosphorylation
GO:0008219	cell death
GO:0016265	death
GO:0008283	cell proliferation
GO:0009893	positive regulation of metabolic process
GO:0048583	regulation of response to stimulus
GO:0001775	cell activation
GO:0001816	cytokine production
GO:0001932	regulation of protein phosphorylation
GO:0060255	regulation of macromolecule metabolic process
GO:0051246	regulation of protein metabolic process
GO:0010604	positive regulation of macromolecule metabolic process
GO:0042127	regulation of cell proliferation

Figure S8. GO term analysis of upregulated pathways in Vsir$^{-/-}$ versus WT DCs was performed.

Figure S9.

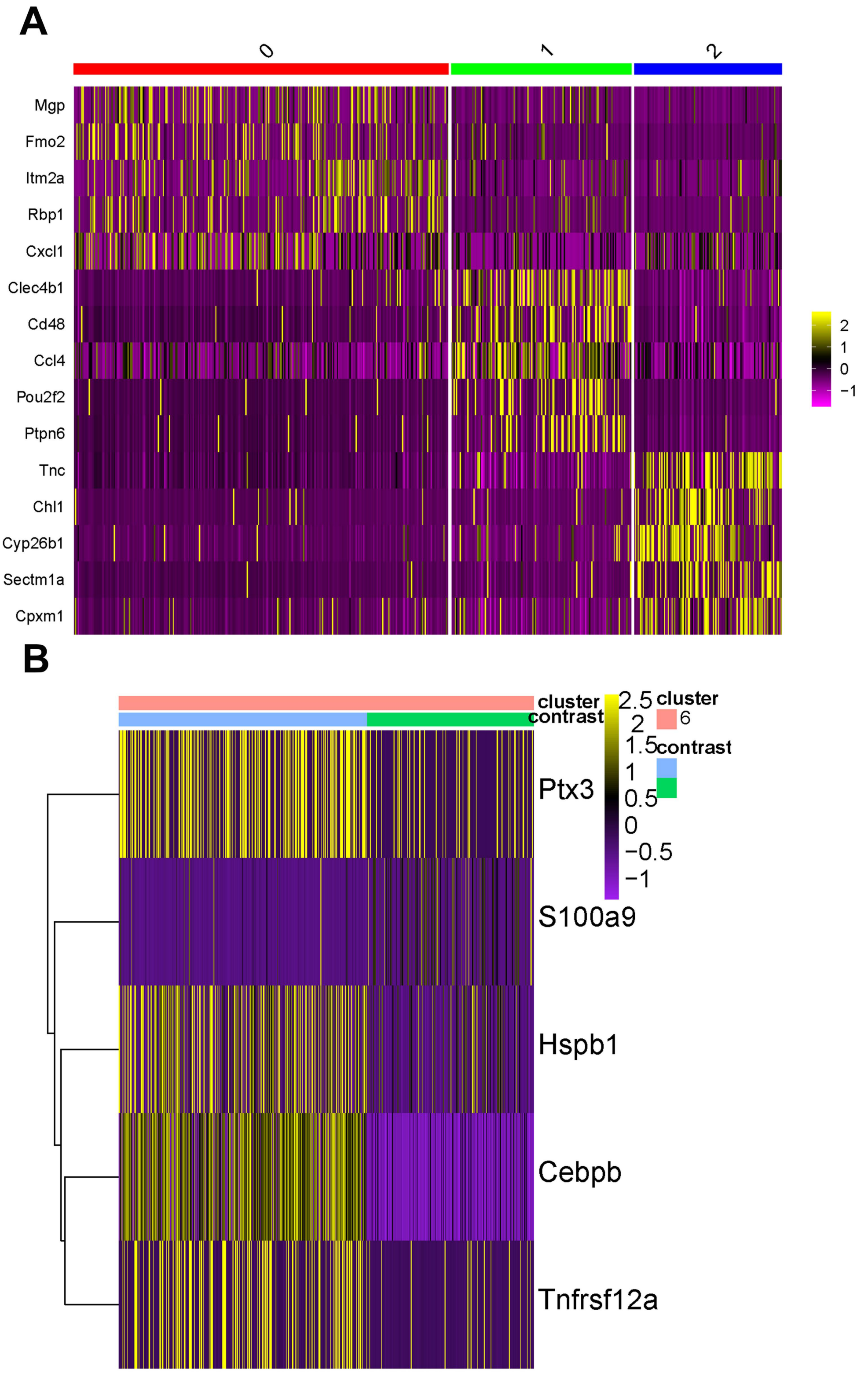

Figure S9. Fibroblast cell clusters were described.

(A) Heatmap showing specific marker genes in each fibroblast cluster. **(B)** Heatmap

of the genes that were at least two-fold upregulated in the fibroblasts from $Vsir^{-/-}$ psoriatic mice compared to that in the fibroblasts isolated from WT psoriatic mouse skin

Figure S10.

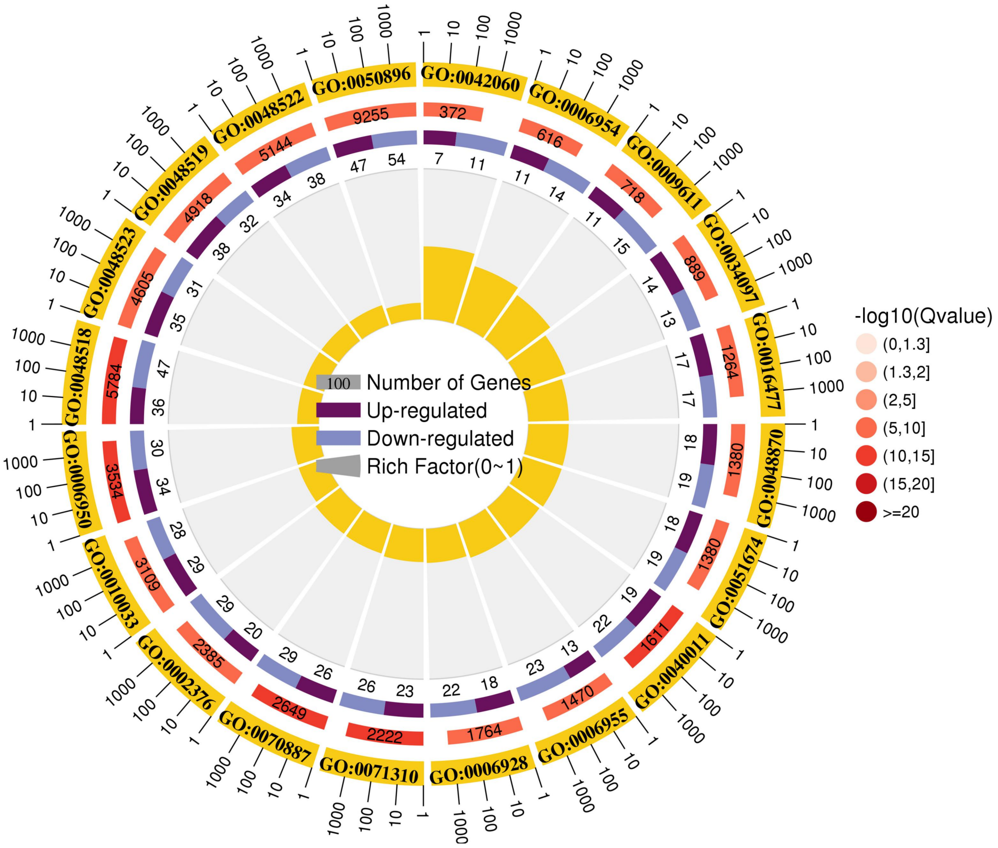

GO ID	Description
GO:0070887	cellular response to chemical stimulus
GO:0006950	response to stress
GO:0048518	positive regulation of biological process
GO:0071310	cellular response to organic substance
GO:0040011	locomotion
GO:0010033	response to organic substance
GO:0048870	cell motility
GO:0051674	localization of cell
GO:0002376	immune system process
GO:0006954	inflammatory response
GO:0009611	response to wounding
GO:0016477	cell migration
GO:0006928	movement of cell or subcellular component
GO:0006955	immune response
GO:0048522	positive regulation of cellular process
GO:0048519	negative regulation of biological process
GO:0034097	response to cytokine
GO:0042060	wound healing
GO:0048523	negative regulation of cellular process
GO:0050896	response to stimulus

Figure S10. GO term analysis of upregulated pathways in Vsir$^{-/-}$ versus WT fibroblasts was performed.

Figure S11.

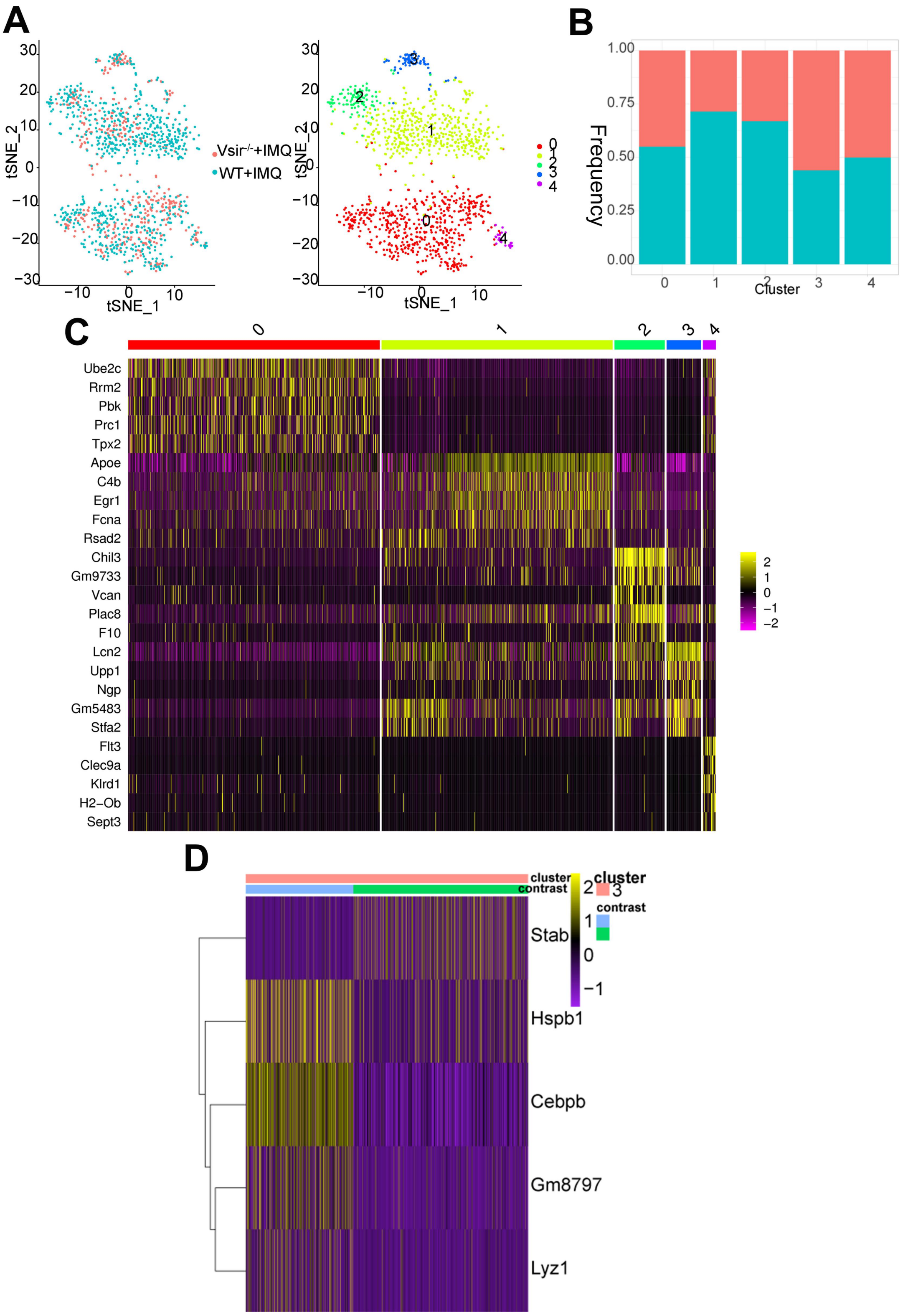

Figure S11. Neutrophil cell clusters were identified.

(A) The sample origin (left panel) and the tSNE plots of 1,261 neutrophils (right panel). **(B)** Bar plots exhibiting the cellular sources for neutrophils subtypes. Blocks represent different subjects and are color-coded by their derived groups defined in figure A. **(C)** Heatmap showing specific marker genes in each neutrophil cluster. **(D)** Heatmap of differentially expressed genes that were at least two-fold upregulated in neutrophil cell clusters from $Vsir^{-/-}$ psoriatic mice compared to neutrophil cell clusters isolated from WT psoriatic mice skin. tSNE, t-distributed stochastic neighbour embedding

Figure S12.

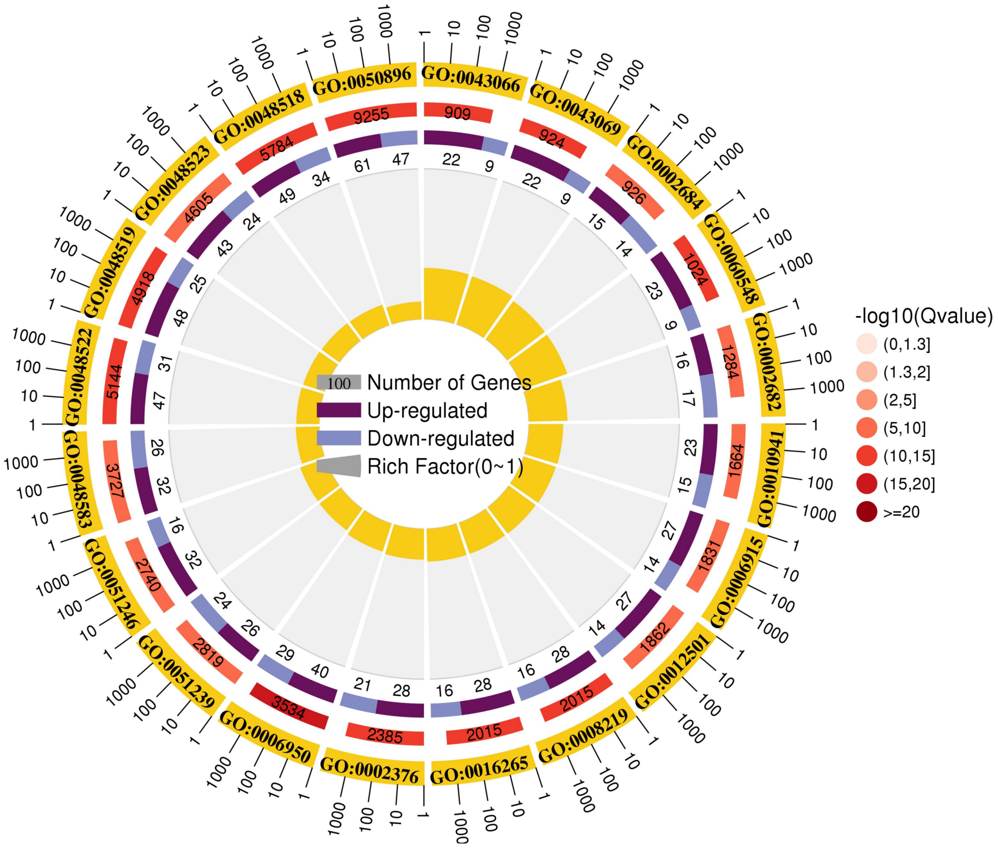

GO ID	Description
GO:0006950	response to stress
GO:0050896	response to stimulus
GO:0048522	positive regulation of cellular process
GO:0048518	positive regulation of biological process
GO:0048519	negative regulation of biological process
GO:0043066	negative regulation of apoptotic process
GO:0043069	negative regulation of programmed cell death
GO:0002376	immune system process
GO:0060548	negative regulation of cell death
GO:0008219	cell death
GO:0016265	death
GO:0006915	apoptotic process
GO:0012501	programmed cell death
GO:0002684	positive regulation of immune system process
GO:0048523	negative regulation of cellular process
GO:0010941	regulation of cell death
GO:0051239	regulation of multicellular organismal process
GO:0002682	regulation of immune system process
GO:0048583	regulation of response to stimulus
GO:0051246	regulation of protein metabolic process

Figure S12. GO term analysis of upregulated pathways of in Vsir$^{-/-}$ versus WT neutrophils was performed.

Figure S13.

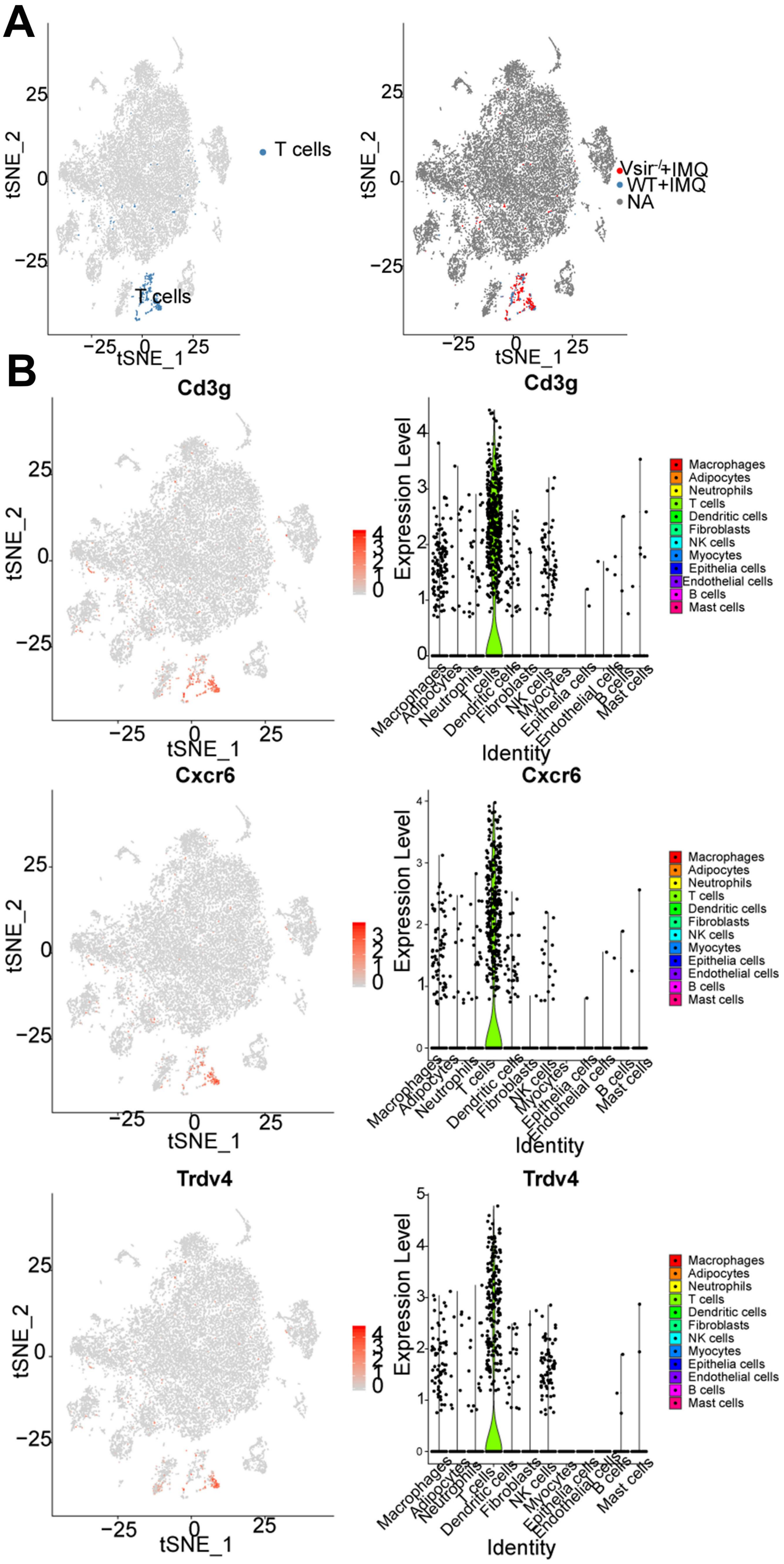
A
T cells
tSNE_1
tSNE_2
Vsir-/-+IMQ
WT+IMQ
NA
B
Cd3g
Cxcr6
Trdv4
Expression Level
Identity
Macrophages
Adipocytes
Neutrophils
T cells
Dendritic cells
Fibroblasts
NK cells
Myocytes
Epithelia cells
Endothelial cells
B cells
Mast cells

Figure S13. T cells were identified.

(A) The tSNE plots of 625 T cells (left panel) and the sample type of origin (right panel). **(B)** tSNE or expression (color bar, TP10K) of key T cell marker genes. tSNE, t-distributed stochastic neighbour embedding.

Figure S14.

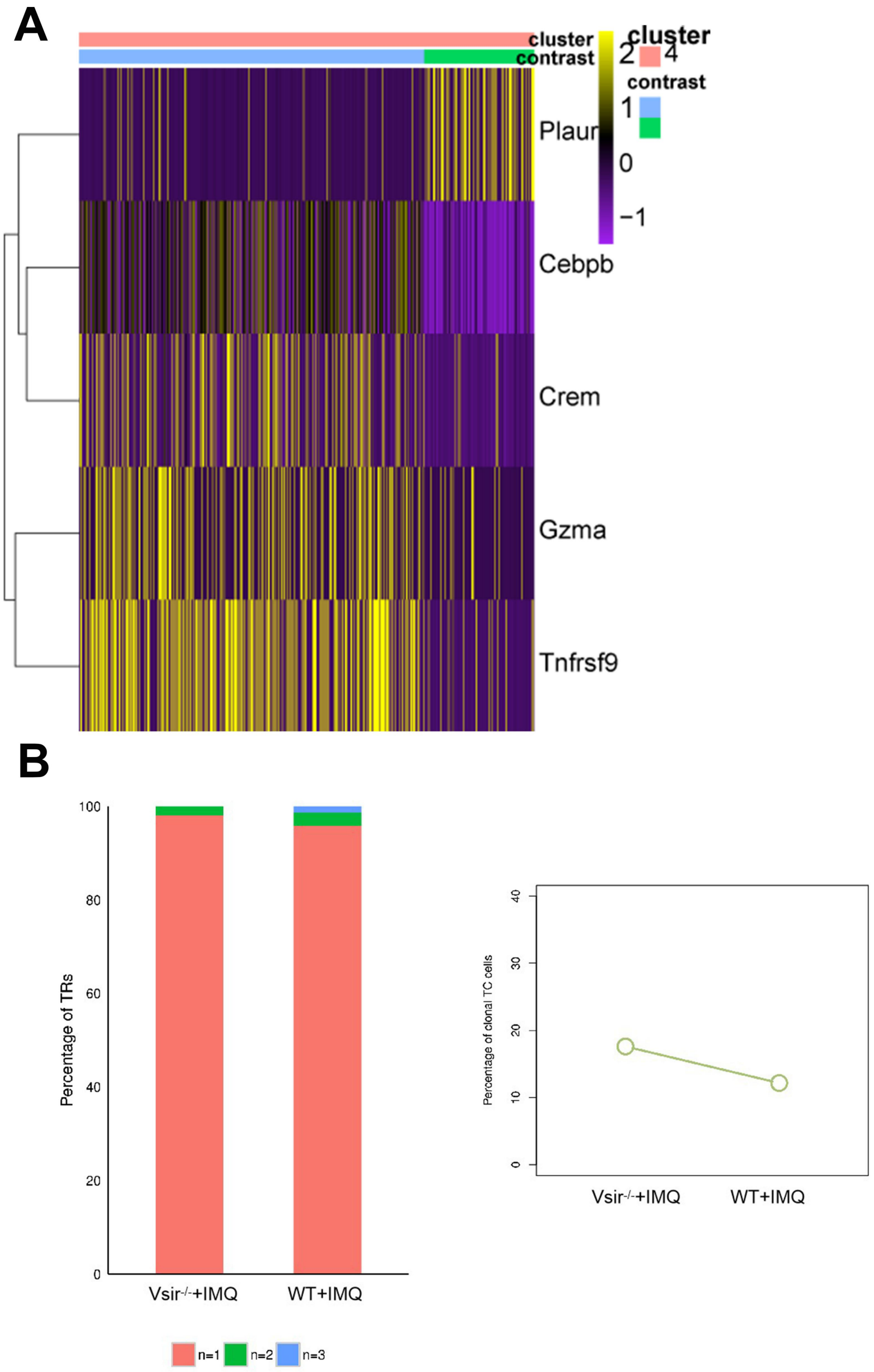

Figure S14. The signature of T cells in Vsir$^{-/-}$ psoriatic mice has been described.

(A) Heatmap of genes that were at least two-fold upregulated in T cells from Vsir$^{-/-}$

psoriatic mice compared to T cells isolated from WT psoriatic mice skin. **(B)** The TCR distribution of T cells across different groups. Unique (n = 1), duplicated (n = 2), and clonal (n ≥ 3) TCRs are labeled with different colors (left panel). The proportions of clonal T cells in two different groups (right panel).

Figure S15.

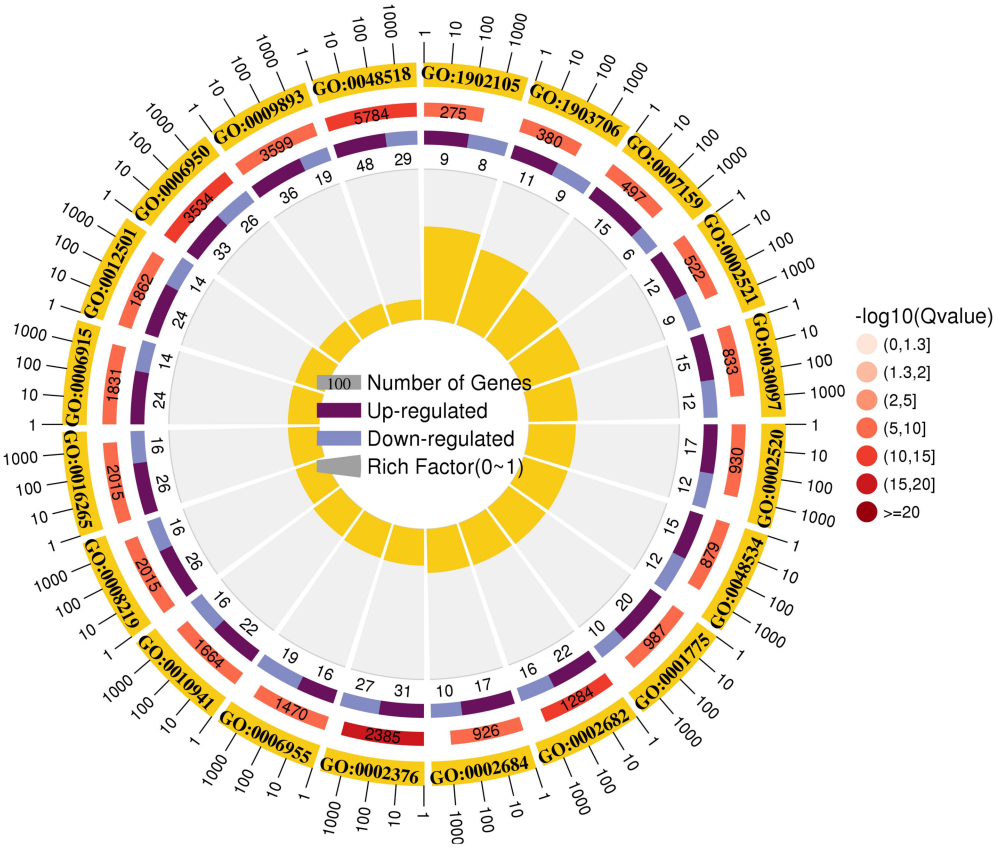

GO ID	Description
GO:0002376	immune system process
GO:0002682	regulation of immune system process
GO:0006950	response to stress
GO:0048518	positive regulation of biological process
GO:1903706	regulation of hemopoiesis
GO:0001775	cell activation
GO:0002520	immune system development
GO:0008219	cell death
GO:0016265	death
GO:0010941	regulation of cell death
GO:0030097	hemopoiesis
GO:1902105	regulation of leukocyte differentiation
GO:0006955	immune response
GO:0007159	leukocyte cell-cell adhesion
GO:0048534	hematopoietic or lymphoid organ development
GO:0002521	leukocyte differentiation
GO:0002684	positive regulation of immune system process
GO:0006915	apoptotic process
GO:0009893	positive regulation of metabolic process
GO:0012501	programmed cell death

Figure S15. GO term analysis of upregulated pathways of in Vsir$^{-/-}$ versus WT T cells were performed.

Figure S16.

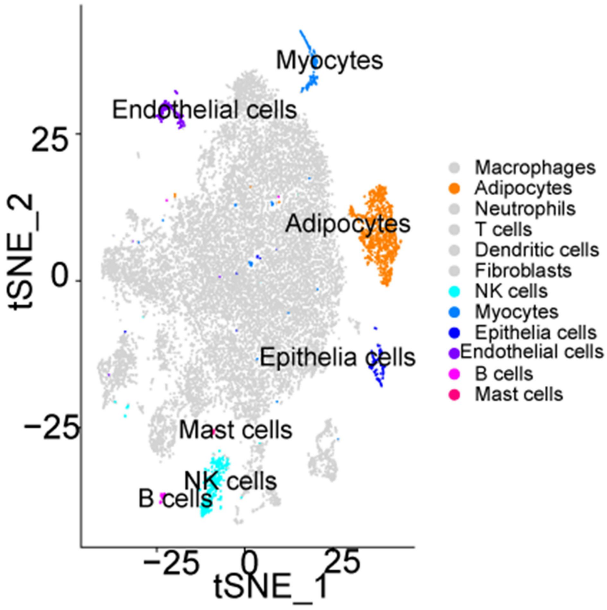

Figure S16. Other clusters in murine psoriasis were defined. The tSNE plot of NK cells, B cells, mast cells, endothelial cells, epithelia cells, myocytes and adipocytes was shown. NK cells, natural killer cells. tSNE, t-distributed stochastic neighbour embedding

Figure S17.

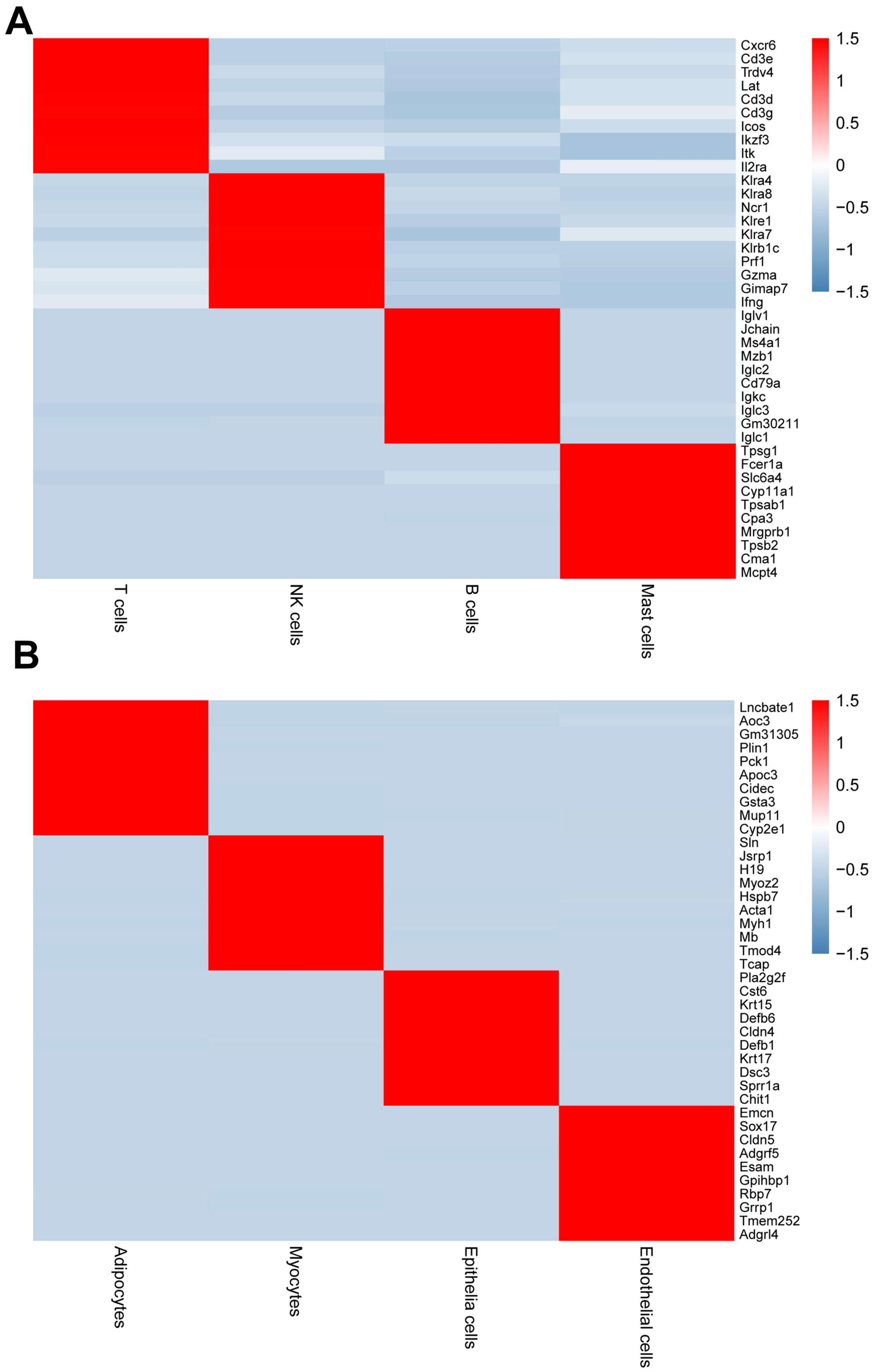

Figure S17. **(A)** Heatmap showing the 10 most upregulated genes (ordered by

decreasing p-value) in T cells, NK cells, B cells and mast cells defined in Figure S16, and selected enriched genes used for biological identification of clusters. **(B)** Heatmap showing the 10 most upregulated genes (ordered by decreasing p-value) in adipocytes, myocytes, epithelia cells and endothelial cells, and selected enriched genes used for biological identification of clusters. NK cells, natural killer cells.

Figure S18.

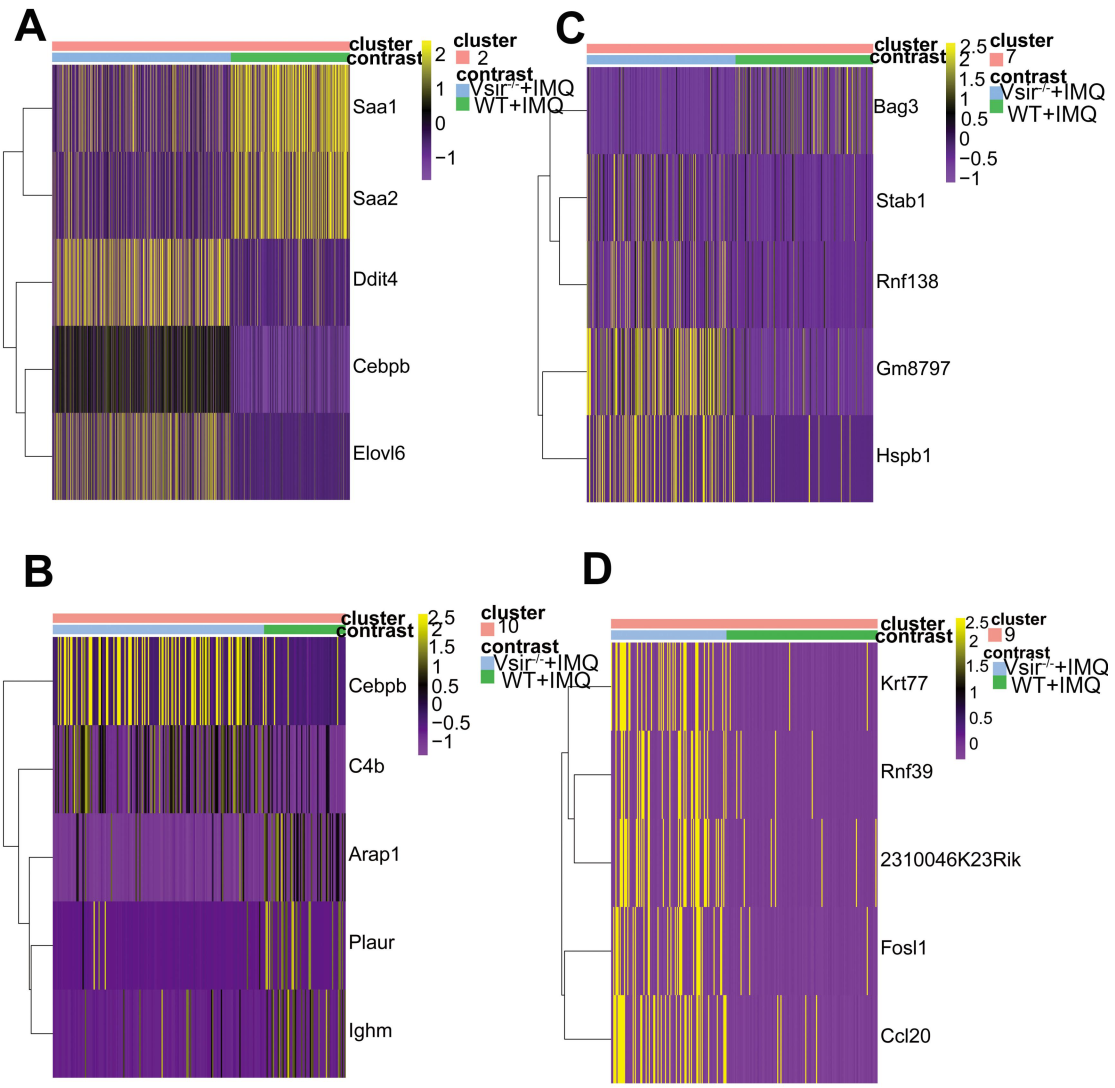

Figure S18. Heatmap showed differentially expressed genes in other cell types. (A-D) Heatmap of genes that were at least two-fold upregulated in adipocytes **(A)**, endothelial cells **(B)**, NK cells **(C)**, epithelia cells **(D)** from Vsir$^{-/-}$ psoriatic mice compared to WT psoriatic mice skin. NK cells, natural killer cells.

Figure S19.

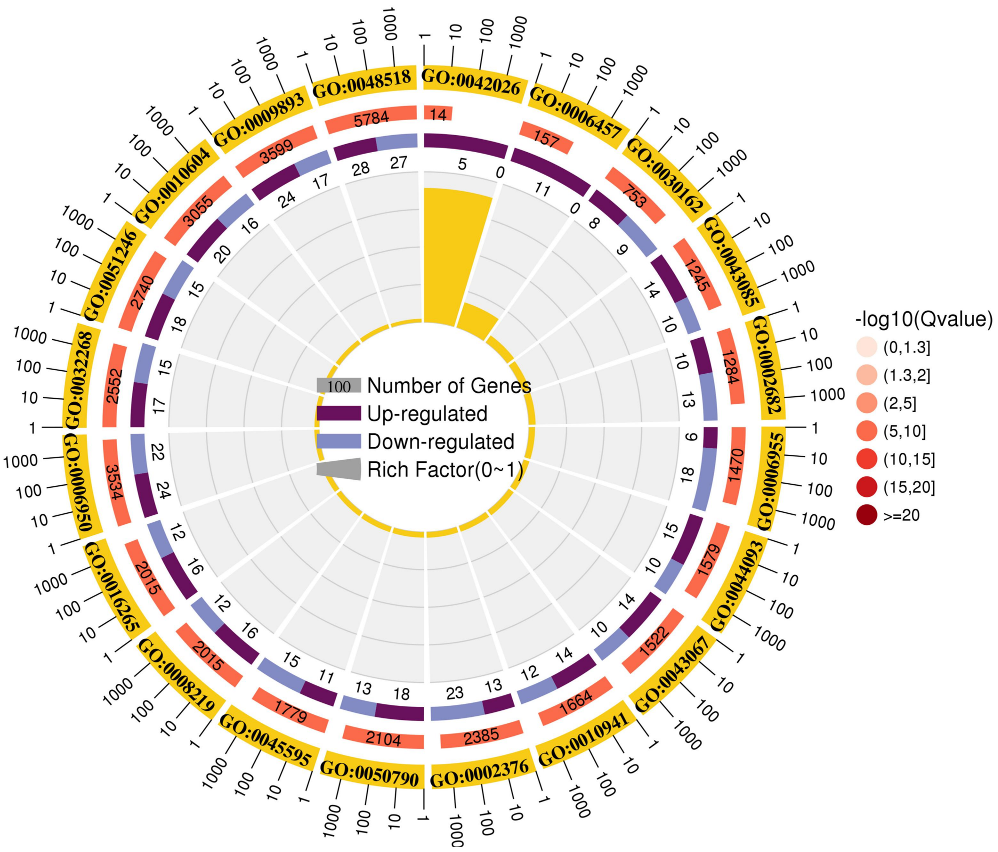

GO ID	Description
GO:0006950	response to stress
GO:0002376	immune system process
GO:0006457	protein folding
GO:0048518	positive regulation of biological process
GO:0043085	positive regulation of catalytic activity
GO:0050790	regulation of catalytic activity
GO:0009893	positive regulation of metabolic process
GO:0042026	protein refolding
GO:0002682	regulation of immune system process
GO:0010941	regulation of cell death
GO:0010604	positive regulation of macromolecule metabolic process
GO:0044093	positive regulation of molecular function
GO:0006955	immune response
GO:0032268	regulation of cellular protein metabolic process
GO:0008219	cell death
GO:0016265	death
GO:0030162	regulation of proteolysis
GO:0051246	regulation of protein metabolic process
GO:0043067	regulation of programmed cell death
GO:0045595	regulation of cell differentiation

Figure S19. GO term analysis of upregulated pathways of in Vsir$^{-/-}$ versus WT NK cells.

Table S1

Table S1 Overview of sequencing data quality metrics for cells extracted from WT and $Vsir^{-/-}$ psoriatic mice.

	WT+IMQ	$Vsir^{-/-}$+IMQ
Estimated number of cells	12,040	11,218
Number of Reads	318,400,966	285,931,105
Valid Barcodes	93.7%	93.2%
Reads Mapped Confidently to Transcriptome	71.8%	69.4%
Reads Mapped Confidently to Exonic Regions	76.5%	73.1%
Reads Mapped Confidently to Intronic Regions	7.2%	6.0%
Reads Mapped Confidently to Intergenic Regions	5.0%	6.4%
Reads Mapped Antisense to Gene	3.9%	2.7%
Sequencing Saturation	73.4%	77.5%
Q30 Bases in Barcode	96.1%	95.0%
Q30 Bases in RNA Read	91.1%	92.4%
Q30 Bases in UMI	95.5%	93.5%
